# Human Organoids Reveal PTEN-Driven Mesendoderm Specification via Retinoic Acid Signaling Suppression

**DOI:** 10.34133/research.1374

**Published:** 2026-08-14

**Authors:** Wuming Wang, Qiqi He, Hong Zhang, See-Wing Chan, Zhiqiang Xiong, Haite Tang, Yue Lv, Xianwei Su, Ya Guo, Yong Fan, Xingguo Liu, Xiuwu Bian, Andrew M. Chan, Hongbin Liu, Gang Lu, Wai-Yee Chan

**Affiliations:** ^1^ School of Biomedical Sciences, Faculty of Medicine, The Chinese University of Hong Kong, Hong Kong SAR, China.; ^2^ CUHK-SDU Joint Laboratory on Reproductive Genetics, School of Biomedical Sciences, The Chinese University of Hong Kong, Hong Kong SAR, China.; ^3^ Key Laboratory for Regenerative Medicine, Ministry of Education, School of Biomedical Sciences, Faculty of Medicine, The Chinese University of Hong Kong, Hong Kong SAR, China.; ^4^ Kunming Institute of Zoology– The Chinese University of Hong Kong (KIZ-CUHK) Joint Laboratory of Bioresources and Molecular Research of Common Diseases, The Chinese University of Hong Kong, Hong Kong SAR, China.; ^5^ CUHK-GIBH CAS Joint Research Laboratory on Stem Cell and Regenerative Medicine, The Chinese University of Hong Kong, Hong Kong SAR, China.; ^6^ Center for Neuromusculoskeletal Restorative Medicine, Hong Kong Science Park, Shatin, New Territories, Hong Kong SAR, China.; ^7^ Shandong Key Laboratory of Reproductive Medicine, Shandong Provincial Hospital affiliated to Shandong First Medical University, Jinan 250012, Shandong, China.; ^8^ Chongqing-Hongkong Healthtech Hub, Jinfeng Laboratory, Chongqing, China.; ^9^ Key Laboratory for the Genetics of Developmental and Neuropsychiatric Disorders (Ministry of Education), Joint International Research Laboratory of Metabolic and Developmental Sciences, School of Life Sciences and Biotechnology, Shanghai Jiao Tong University, Shanghai 200240, China.; ^10^ Department of Obstetrics and Gynecology, Guangdong Provincial Key Laboratory of Major Obstetric Diseases, Guangdong Provincial Clinical Research Center for Obstetrics and Gynecology, Guangdong-Hong Kong-Macao Greater Bay Area Higher Education Joint Laboratory of Maternal-Fetal Medicine, The Third Affiliated Hospital, Guangzhou Medical University, Guangzhou 510150, China.; ^11^ Joint School of Life Sciences, Guangzhou Institutes of Biomedicine and Health, Chinese Academy of Sciences; Guangzhou Medical University, Guangzhou, China.; ^12^ State Key Laboratory of Reproductive Medicine and Offspring Health, Center for Reproductive Medicine, Institute of Women, Children and Reproductive Health, Shandong University, 250012, China.; ^13^ Hong Kong Branch of CAS Center for Excellence in Animal Evolution and Genetics, The Chinese University of Hong Kong, Hong Kong SAR, China.; ^14^ The Chinese University of Hong Kong - University of Southampton Joint Laboratory for Stem Cell and Regenerative Medicine, School of Biomedical Sciences, The Chinese University of Hong Kong, Hong Kong SAR, China.

## Abstract

During early embryogenesis, epiblast cells ingress through the primitive streak (PS) to adopt a mesendoderm (MES) fate, giving rise to both mesodermal and endodermal lineages. Robust systems that accurately recapitulate MES specification—and thereby enable a comprehensive understanding of cell fate transitions during PS formation—remain limited. Here, we describe 3-dimensional MES organoids derived from human induced pluripotent stem cells that faithfully model in vivo human MES specification, particularly from the anterior PS. MES specification requires *PTEN* expression, as *PTEN* loss profoundly impairs mesoderm and definitive endoderm derivatives. Integrative multi-omics analyses—including transcriptomics, chromatin accessibility, metabolomics, and phosphoproteomics—on wild-type and *PTEN^−/−^* MES cells revealed that *PTEN* suppresses retinoic acid (RA) signaling during MES commitment. Furthermore, we identified the RA-degrading enzyme *CYP26A1* as a downstream effector of *PTEN*. Notably, while excessive RA induced by *PTEN* ablation is detrimental to MES cell generation, physiological levels of RA are necessary. Collectively, the human MES organoids offer a valuable model for dissecting early human development, and our findings identify *PTEN* as a key regulator of MES fate commitment through inhibition of RA signaling.

## Introduction

The formation of the primitive streak (PS)—a transient, thickened region of the epiblast—marks a pivotal event during gastrulation. Within the PS, epiblast cells acquire distinct fates in a spatially and temporally regulated manner. Cells ingressing into the anterior PS (APS) adopt a mesendoderm (MES) identity, coexpressing lineage-specifying transcription factors such as *EOMES*, *SOX17*, *GSC*, *FOXA2*, and *LHX1* [1]. These MES cells then undergo epithelial–mesenchymal transition (EMT), leading to differentiation into the mesoderm and definitive endoderm (DE) [2]. In contrast, cells from the posterior PS (PPS) contribute predominantly to the extraembryonic and somitic mesoderm [3].

Studying early human development poses considerable technical and ethical challenges. Recently, human induced pluripotent stem cell (hiPSC)-derived models, such as blastoids and gastruloids, have emerged as promising tools for investigating early gastrulation events [4–7]. However, these models primarily recapitulate pre-implantation processes, making it challenging to trace mesodermal and endodermal specification during post-implantation stages. Assembloids have been developed to support mesodermal and endodermal lineage differentiation. Co-development of central and peripheral neuronal systems with trunk MES generates elongating multi-lineage organized gastruloids [8]. Co-development of mesoderm and endoderm enables organotypic vascularization in lung and gut organoids [9]. Despite these advances, a dedicated MES organoid model is valuable for elucidating the co-development of mesodermal and endodermal lineages.

Canonical signaling pathways—including Nodal, BMP, and WNT—are central to PS induction and MES cell formation in both embryos and iPSCs [10–12]. However, BMP4 also promotes trophoblast differentiation, complicating lineage isolation, and both BMP4 and WNT support mouse embryonic stem cell self-renewal [13,14], highlighting the complexity of PS regulation. Mouse epiblast stem cells have been shown to recapitulate APS transcriptional and functional features [15], and APS fate can also be induced in hiPSCs via YAP1 modulation [16]. Epigenetically, the chromatin regulator *Whsc1* facilitates both pluripotency exit and MES specification [17], while *SMYD2* promotes MES generation by activating key lineage genes [18]. Nonetheless, the precise mechanisms governing MES specification and APS organization remain incompletely understood.

In this study, we characterized a human MES organoid that models core features of MES specification. These organoids can be further differentiated into DE and mesodermal lineages, offering a tractable platform for studying early lineage commitment. Many congenital defects and spontaneous miscarriages are driven by genetic abnormalities, including mutations in *PTEN* [19–22]. *PTEN* exhibits an expression pattern comparable to that of MES-specific markers during MES organoid formation. We demonstrate that *PTEN* ablation disrupts MES specification in vitro and compromises embryonic development in vivo. Our results indicate that *PTEN* facilitates MES formation by modulating retinoic acid (RA) signaling via *CYP26A1*. These findings advance our understanding of human APS formation and suggest that defective MES commitment may lead to the embryonic lethality observed in *Pten*-deficient models [23], potentially offering mechanistic insight into the etiology of miscarriage.

## Results

### Generation of MES organoids from hiPSCs

**Fig. 1.**
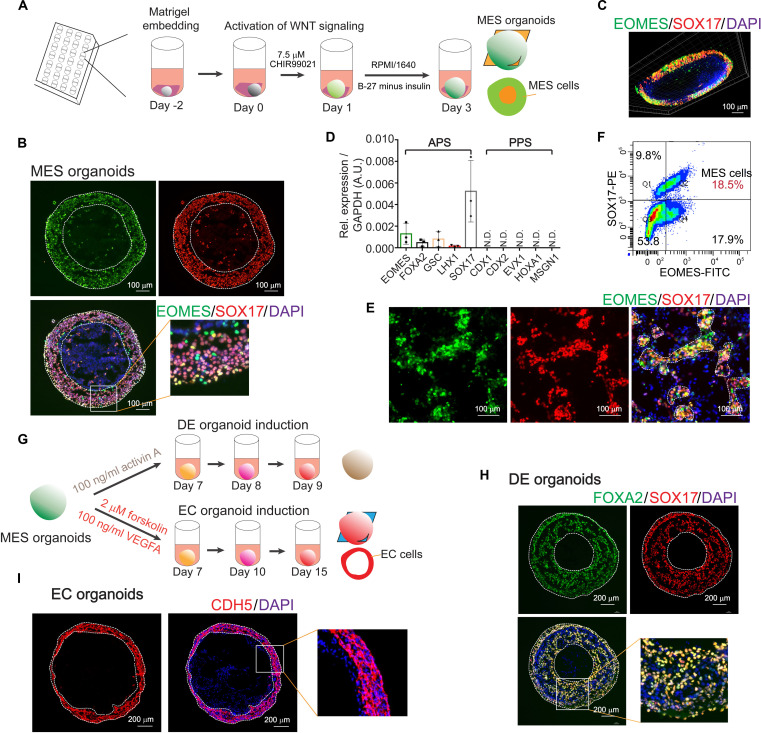
MES organoids recapitulate APS features and differentiate into DE and EC organoids. (A) Schematic overview of the MES organoid generation protocol, wherein individual hiPSC aggregates are embedded in Matrigel and differentiated via WNT pathway activation. (B) IF staining of cryosectioned MES organoids confirming EOMES and SOX17 coexpression. Scale bar, 100 μm. (C) Longitudinal section of a representative MES organoid showing abundant MES cells coexpressing SOX17 and EOMES. Scale bar, 100 μm. (D) Quantitative PCR analysis of APS and PPS marker gene expression, demonstrating enrichment of APS markers. (E) Single-cell dissociation of MES organoids followed by 24-h reculture to assess cellular behavior. Scale bar, 100 μm. (F) Flow cytometric quantification of MES cells from dissociated organoids. (G) Differentiation of MES organoids into DE and EC lineages. (H) IF staining of DE organoid cryosections derived from MES organoids, showing robust expression of DE markers SOX17 and FOXA2. Scale bar, 200 μm. (I) IF staining of DE organoid cryosections demonstrating expression of the EC marker CDH5. Scale bar, 200 μm.

WNT and transforming growth factor-β (TGF-β) signaling are known to induce in vitro models of PS formation using embryonic stem cells [24]. In this study, we established a human MES organoid model by modifying a previously described protocol [25] (Fig. 1A). The resulting organoids developed a prominent layer of MES cells coexpressing *EOMES* and *SOX17*, closely recapitulating in vivo MES cell emergence during PS development (Fig. 1B). Three-dimensional (3D) multiscale imaging further revealed a distinct MES-positive cell layer within the organoids (Fig. 1C and Movie S1). Consistent with an APS-like identity, our MES organoids expressed hallmark MES genes (*SOX17*, *EOMES*, *GSC*, *FOXA2*, *LHX1*) but lacked PPS-specific transcripts such as *CDX1*, *CDX2*, *EVX1*, *HOXA1*, and *MSGN1* (Fig. 1D). We further validated these findings using 2 additional iPSC lines, both of which generated MES organoids with a similar APS-like identity, characterized by a high abundance of MES cells and robust expression of APS marker genes (Fig. S1A and B). These data indicate that the MES organoids closely model APS cells in vivo*.*

Temporal analysis showed significant up-regulation of *EOMES* and *SOX17* expression on days 2 and 3 of differentiation (Fig. S1C and D). Whole-mount immunofluorescence (IF) staining also confirmed the abundance of MES cells coexpressing *EOMES* and *SOX17* within the organoids (Fig. S1E). Upon dissociation and 24-h culture, MES-derived cells exhibited robust migratory behavior, converging into cell clusters (Fig. 1E). Flow cytometric analysis quantified MES cells as comprising approximately 18.5% of the organoid population (Fig. 1F)*.* This proportion was lower than that estimated by IF staining (Fig. 1B and F), likely reflecting the preferential surface localization of MES cells and inter-organoid variability in their abundance (Fig. S3B).

MES cells possess bipotentiality toward mesodermal and endodermal lineages. We leveraged this capacity by differentiating MES organoids into DE organoids, which exhibited specific expression of *SOX17* and *FOXA2* (Fig. 1G and H), indicating potential for pancreatic and hepatic lineage development. To examine mesodermal potential, MES organoids were treated with vascular endothelial growth factor (VEGF) and forskolin, which induced endothelial cell (EC) differentiation and the formation of EC organoids (Fig. 1G). These were characterized by a CDH5-positive EC ring (Fig. 1I). Bright-field and IF imaging revealed a chamber-like morphology (Fig. S1F), and dissociation confirmed enrichment of ECs (Fig. S1G and Movie S2). Together, these results highlight the developmental versatility of MES organoids and their utility in modeling mesodermal and endodermal lineage specification in vitro.

### Single-cell RNA sequencing reveals MES cell fate determination

**Fig. 2.**
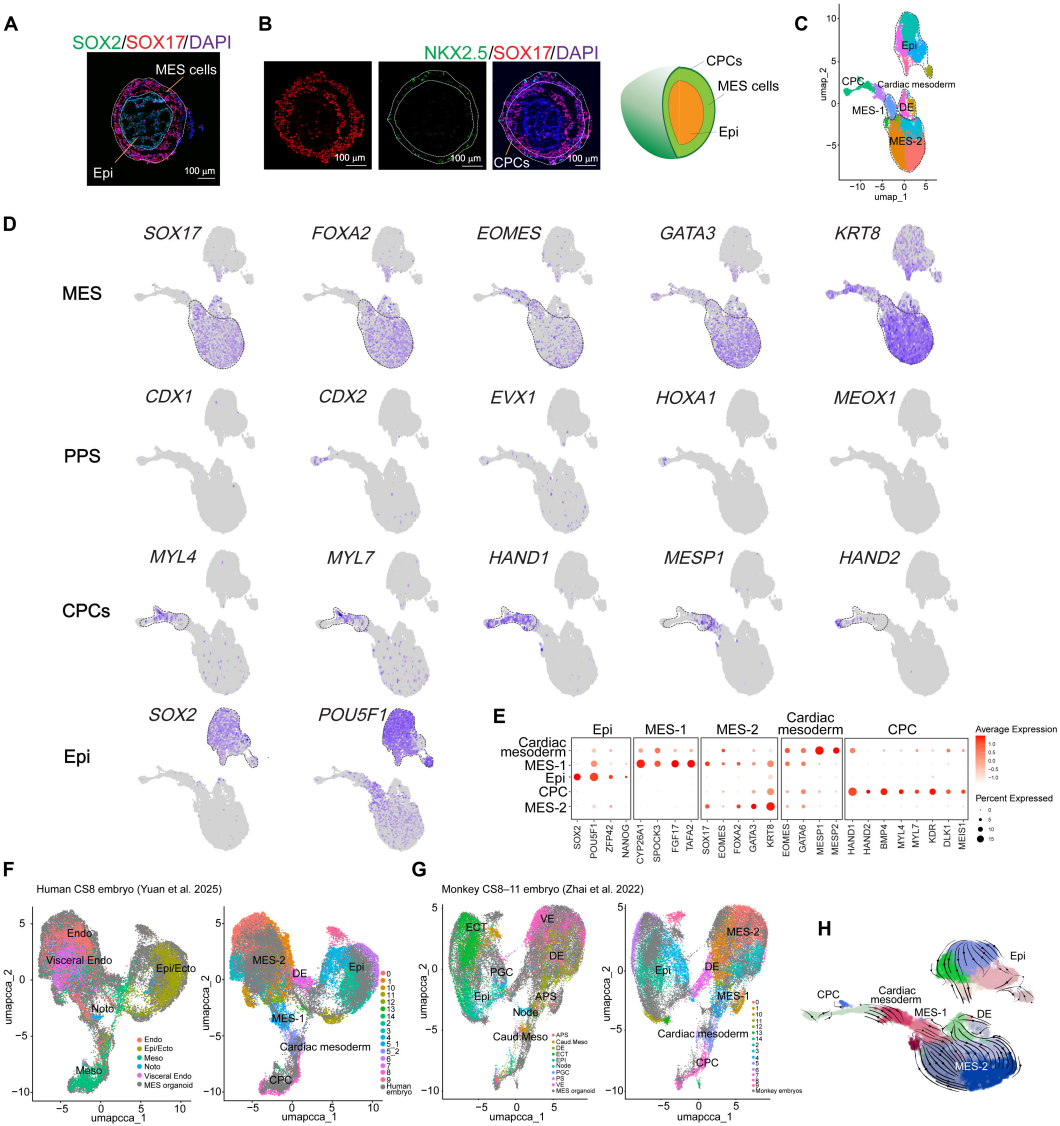
Single-cell transcriptomic profiling of MES organoids. (A) IF staining showing cellular diversity in MES organoids, including out-layer MES cells and inner-layer epiblast (Epi) cells. Scale bar, 100 μm. (B) Detection of NKX2.5-positive cells indicating the presence of CPCs on the surface of MES organoids. Scale bar, 100 μm. (C) UMAP visualization of scRNA-seq data from MES organoids at day 3, revealing distinct cellular clusters including Epi, cardiac mesoderm, CPC, definitive endoderm (DE), MES-1, and MES-2. (D) UMAP feature plots depicting the expression of lineage-specific marker genes corresponding to MES, PPS, CPCs, and Epi populations. (E) Dot plot summarizing the expression profiles of lineage markers across Epi, MES-1, MES-2, cardiac mesoderm, and CPC clusters. (F) UMAPs showing the integration of MES organoids with reference human CS8 embryo-originated cells, demonstrating strong transcriptional similarity. Endo, endoderm; Epi/Ecto, epiblast/ectoderm; Gast/PS, gastrulating cells in the primitive streak; Meso, mesoderm; Noto, notochord; Visceral Endo, visceral endoderm. (G) UMAPs showing the integration of MES organoids with reference monkey CS8-11 embryo-originated cells. APS, anterior primitive streak; Caud. Meso, caudal mesoderm; ECT, ectoderm; PGCs, primordial germ cells; PS, primitive streak; VE, visceral endoderm. (H) RNA velocity analysis overlaid on UMAP, revealing dynamic lineage trajectories within the MES organoids.

Differentiation of iPSCs into MES organoids yields a heterogeneous population of cell types that recapitulate aspects of early embryonic development. We performed single-cell RNA sequencing (scRNA-seq) to delineate the cellular composition of MES organoids. A substantial fraction of cells expressed *SOX2*, a canonical epiblast (Epi) marker (Fig. 2A and C), consistent with Uniform Manifold Approximation and Projection (UMAP) clustering that revealed a distinct Epi population coexpressing *SOX2* and *POU5F1* (Fig. 2D). A minor subset of cardiac progenitor cells (CPCs) was observed predominantly in the organoid periphery (Fig. 2B), expressing markers such as *HAND1*, *HAND2*, *MYL4*, *MYL7*, and *MESP1* (Fig. 2D).

MES cell clusters were identified based on expression of *EOMES*, *SOX17*, *FOXA2*, *GATA3*, and *KRT8*, aligning with quantitative polymerase chain reaction (qPCR) and IF staining results (Fig. 2D and Fig. 1B to D). These clusters lacked PPS-specific markers (*CDX1*, *CDX2*, *EVX1*, *HOXA1*, *MEOX1*), confirming their APS identity (Figs. 1D and 2D). A bubble plot was further used to confirm these lineage assignments (Fig. 2E), while the violin plots show consistent marker expression across cell clusters (Fig. S2C and D). Particularly, we observed a population of MES-1 expressing both mesenchymal markers (*CYP26A1*, *SPOCK3*, *FGF17*, *TAFA2*) and MES markers (*EOMES*, *SOX17*, *FOXA2*, *GATA3*, *KRT8*), enhancing their MES identity (Fig. 2E).

To validate the identity of MES organoid-derived lineages, we compared our single-cell transcriptomes to those from human Carnegie stage 8 (CS8) embryos [26,27]. Several cell lineages showed striking similarities, including Epi and mesoderm (cardiac mesoderm and CPC) (Fig. 2F). The MES-1 population in MES organoids exhibited strong transcriptional similarity to notochord (Noto) cells, which originate from APS during early gastrulation (Fig. 2F) [28]. Most MES-2 cells resembled visceral endoderm (VE) and DE from human embryos, likely due to their exhibiting properties of both mesoderm and endoderm (Fig. 2F). Integrative analysis with monkey embryo transcriptomes further revealed prominent APS identity in the MES-1 population, alongside similarities in Epi and caudal mesoderm (Caud. Meso) lineages (Fig. 2G) [29]. As observed in human embryos, MES-2 cells aligned closely with VE and DE in monkey embryos (Fig. 2G). To provide a more quantitative assessment of developmental mapping, we incorporated a Sankey diagram illustrating the cellular composition of MES organoids in comparison with human and monkey embryos (Fig. S2E and F). This analysis demonstrates that MES organoids recapitulate a proportion of well-characterized cell types, including VE, APS, Caud. Meso, primordial germ cells (PGCs), node, Epi, PS, DE, and ectoderm (ECT) (Fig. S2F).

RNA velocity and diffusion mapping identified a principal trajectory from the MES-1 cluster to MES-2, indicating that MES-1 represents APS cells that progress to MES-2 with stronger endodermal identity (Fig. 2H). Compared to 2D MES cells, MES organoids provide an ideal system for tracing the derivation and cell fates of these populations, particularly APS cells. These results validate the fidelity of MES organoids as a model for early human development and suggest their utility in uncovering mechanisms relevant to implantation failure and miscarriage.

### MES organoids reveal *PTEN*’s role in driving MES lineage specification

**Fig. 3.**
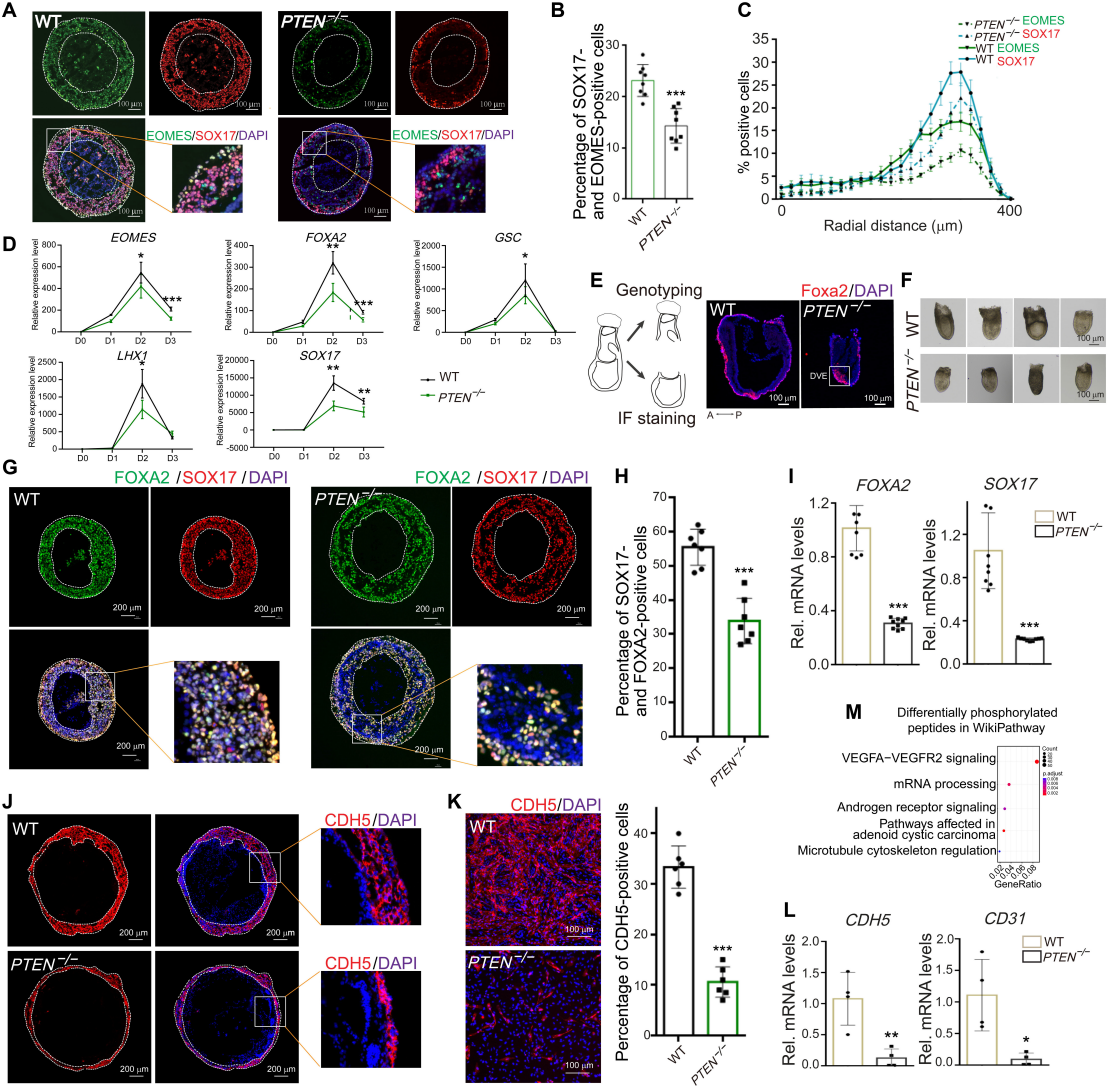
*PTEN* is essential for MES organoid organization and early embryonic development. (A) *PTEN^−/−^* MES organoids show a marked reduction in SOX17- and EOMES-positive MES cells. Scale bar, 100 μm. (B) Proportion of MES cells coexpressing SOX17 and EOMES in WT and *PTEN^−/−^* MES organoids (*n* = 8, ***P < 0.001). (C) Radial profiles of the proportion of MES cells. (D) qPCR analysis reveals dynamic changes in the expression of MES markers, including *GSC*, *FOXA2*, *SOX17*, *LHX1*, and *EOMES*, between WT and *PTEN^−/−^* organoids (*n* = 3, *P < 0.05; **P < 0.01; ***P < 0.001). (E) IF staining shows that Foxa2-positive cells are predominantly localized within the distal region of the visceral endoderm in *PTEN^−/−^* mouse embryos. Scale bar, 100 μm. (F) Developmental progression is impaired in *PTEN^−/−^* mouse embryos compared to WT at E7.5. Scale bar, 100 μm. (G) Cryosection of a representative DE organoid demonstrates the essential role of *PTEN* in DE differentiation. Scale bar, 200 μm. (H) Proportion of DE cells coexpressing SOX17 and FOXA2 in WT and *PTEN^−/−^* DE organoids (*n* = 7, ***P < 0.001). (I) mRNA levels of DE markers FOXA2 and SOX17 are significantly reduced in *PTEN^−/−^* DE organoids (*n* = 7, ***P < 0.001). (J) *PTEN^−/−^* EC organoids exhibit impaired EC ring formation. Scale bar, 200 μm. (K) Representative images of dissociated ECs from WT and *PTEN^−/−^* organoids. Scale bar, 100 μm (*n* = 6, ***P < 0.001). (L) *PTEN* deficiency significantly reduces mRNA expression of EC markers CDH5 and CD31 (*n* = 4, *P < 0.05; **P < 0.01). (M) Phosphoproteomic profiling indicates enrichment of VEGFA–VEGFR2 signaling in WT versus *PTEN^−/−^* EC organoids.

*Pten* is indispensable for embryogenesis; *Pten*-null embryos exhibit lethality at embryonic day 7.5 (E7.5) [23]. Clinically, *PTEN* dysregulation has been linked to spontaneous miscarriage [20–22], further underscoring its crucial role in early development. However, the role of *PTEN* during early human development remains incompletely understood. Our data revealed that PTEN protein expression followed a temporal pattern similar to that of MES marker genes during MES specification, with both peaking at day 2 (Fig. S1D, H, and I). These findings suggest that *PTEN*, classically characterized as a tumor suppressor, may also participate in MES specification. Using CRISPR/Cas9, we generated *PTEN^−/−^* iPSCs (Fig. S3A) and observed that loss of *PTEN* significantly impaired MES formation. MES organoids derived from *PTEN^−/−^* cells exhibited a marked reduction in *SOX17*- and *EOMES*-positive cells (Fig. 3A and B and Fig. S3B). The MES cell ring was also visibly constricted in *PTEN^−/−^* organoids (Fig. 3C). Correspondingly, expression of key MES markers (*EOMES*, *FOXA2*, *SOX17*, *GSC*, *LHX1*) was significantly down-regulated in *PTEN*-deficient MES organoids (Fig. S3C). We further validated the role of *PTEN* in MES organoid formation using an additional iPSC line, AICS-0060-027, and observed results consistent with those obtained in the AG28869 line, including a reduced proportion of MES-positive cells and decreased expression of MES marker genes (Fig. S3D to F). Temporal expression analysis further revealed that *PTEN* deficiency markedly suppressed MES gene expression at day 2 during organoid formation (Fig. 3D), aligning precisely with the observed peak in PTEN protein levels (Fig. S1H and I) and thereby confirming *PTEN*’s role in promoting MES specification.

To explore the in vivo relevance of these findings, we intercrossed *PTEN^+/−^* mice and collected wild-type (WT) and *PTEN^−/−^* embryos at E7.5, prior to lethality. *Foxa2*, in addition to marking APS cells and DE progenitors, also orchestrates the patterning of the PS epiblast [30]. In *PTEN^−/−^* embryos, *Foxa2*-positive cells were concentrated within the distal VE—the precursor to the anterior VE (AVE)—suggesting impaired cell migration (Fig. 3E). This observation aligns with previous findings that *PTEN* is necessary for proper AVE migration, which determines PS positioning [31]. Moreover, *PTEN^−/−^* embryos were significantly smaller than WT counterparts, indicating delayed or defective embryonic development (Fig. 3F). These developmental defects likely underlie the early embryonic lethality observed in *Pten*-deficient mice [19].

### The generation of mesodermal and endodermal lineages is impaired by *PTEN* ablation

Gastrulation is characterized by the formation of the PS, where MES cells emerge and give rise to DE and mesoderm lineages [16]. DE arises approximately 8 to 10 h after the onset of gastrulation from the APS [32]. Building on our demonstration that MES organoids can develop into DE organoids (Fig. 1H), we investigated the role of *PTEN* in DE specification using our 3D organoid system. *PTEN* ablation significantly impaired DE generation, as evidenced by decreased *SOX17- and FOXA2*-positive cells (Fig. 3G and H) and reduced expression of DE markers (Fig. 3I).

To corroborate these findings in a 2D model, hiPSCs were directed toward DE cell differentiation, yielding *SOX17*- and *FOXA2*-positive cells under standard conditions. However, *PTEN* knockout markedly reduced the proportion of DE cells, as shown by IF staining (Fig. S3G and H), qPCR (Fig. S3I), and flow cytometric analysis (Fig. S3J). Transcriptomic analysis linked the *PTEN*-dependent DE defects to diabetes-related pathways (Fig. S3K). Notably, *PTEN* ablation led to diffuse nuclear localization of *SOX17* and *FOXA2* proteins (Fig. S3G), suggesting possible nuclear fragmentation, consistent with previous studies showing that nuclear PTEN maintains chromosomal integrity [33]. Together, these results demonstrate that *PTEN* is involved in MES-derived DE cell specification.

*PTEN* deletion also significantly impaired cardiac mesoderm development (Fig. S4). Both IF staining and flow cytometric analysis revealed a decrease in the proportions of *GATA4*- and *NKX2.5*-positive cells in the *PTEN^−/−^* group (Fig. S4A and B). Using a defined protocol for generating human cardiac organoids (hCOs) [25], we observed severely compromised hCO, as evidenced by diminished *NKX2.5*-positive cell populations and decreased mRNA expression levels of CPC markers (*NKX2.5*, *MESP1*, *MESP2*) in the *PTEN^−/−^* cells (Fig. S4C to E), consistent with our previous report implicating *PTEN* in cardiogenesis [34].

ECs, which originate from mesodermal derivatives, form through vasculogenesis in both embryonic and extraembryonic tissues [35]. When MES organoids were directed toward EC differentiation via VEGF and forskolin (Fig. 1G and I), *PTEN* ablation led to a marked reduction in EC ring formation (Fig. 3J and Fig. S5A). Dissociation and replating of EC organoids further revealed significantly fewer ECs in the *PTEN^−/−^* group (Fig. 3K). Correspondingly, mRNA levels of EC markers *CDH5* and *CD31* were significantly reduced (Fig. 3L). Phosphoproteomic profiling of the kinase tree revealed perturbation of the VEGFA–VEGFR2 (VEGF receptor 2) signaling pathway (Fig. 3M and Fig. S5B), underscoring the role of *PTEN* in EC specification from MES organoids.

### *PTEN* promotes MES cell generation and collective migration via its protein phosphatase activity

**Fig. 4.**
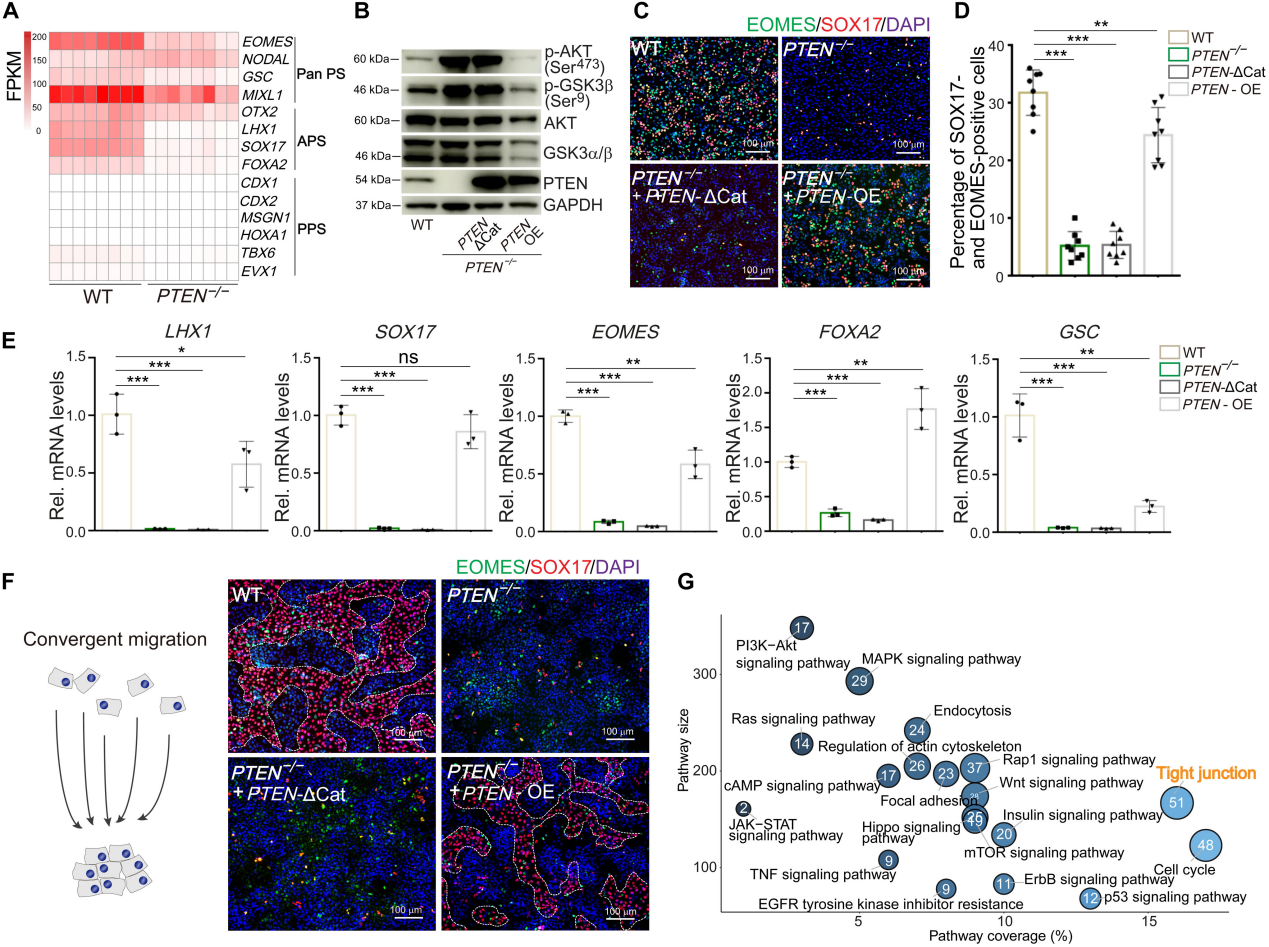
PTEN regulates MES cell generation and migration through its phosphatase activity. (A) Heatmap showing reduced expression of pan-PS and APS markers in *PTEN^−/−^* cells, while no expression in both WT and *PTEN^−/−^* MES cells. (B) Western blot analysis confirming loss of phosphatase activity in the catalytically inactive *PTEN* mutant (*PTEN*-ΔCat). (C and D) PTEN-ΔCat fails to rescue MES generation in *PTEN^−/−^* iPSCs. Scale bar, 100 μm (*n* = 8, **P < 0.01; ***P < 0.001). (E) MES marker gene expression remains suppressed in *PTEN^−/−^* cells expressing *PTEN*-ΔCat (*n* = 3, *P < 0.05; **P < 0.01; ***P < 0.001). (F) Convergent migration of MES cells is impaired in *PTEN^−/−^* cells and is not rescued by PTEN-ΔCat, indicating a requirement for PTEN’s enzymatic activity. Scale bar, 100 μm. (G) Pathway annotation of phosphoproteins in the library using Kyoto Encyclopedia of Genes and Genomes (KEGG) database. Phosphoproteomic analysis reveals enrichment of kinases involved in tight junction signaling, a pathway essential for coordinated cell migration. Circle size represents phosphosite coverage; the *x* and *y* axes indicate protein coverage in the pathway and protein size, respectively. Numbers within circles denote the count of phosphosites covered per pathway, while blue color intensity reflects pathway coverage percentage.

To comprehensively understand *PTEN*’s roles in MES specification and decipher the underlying regulatory mechanism, we employed a 2D MES differentiation protocol. As expected, *PTEN^−/−^* iPSCs showed a substantial reduction in MES cells coexpressing *EOMES* and *SOX17* (Fig. S6A to C). Rescue experiments demonstrated that *PTEN* overexpression (*PTEN*-OE) in the *PTEN^−/−^* iPSCs restored MES cell numbers to WT levels (Fig. S6A and B) and re-established expression of *EOMES*, *LHX1*, *SOX17*, *FOXA2*, and *GSC* (Fig. S6C). Bulk RNA sequencing further confirmed down-regulation of MES-specific genes in *PTEN^−/−^* MES cells, without detectable expression of PPS markers (Fig. 4A), consistent with observations made in MES organoids (Figs. 1D and 2D).

PTEN is best known for its lipid phosphatase activity, which antagonizes phosphatidylinositol 3-kinase (PI3K)/AKT signaling [36]. To probe whether this activity underlies its role in MES specification, we treated *PTEN^−/−^* iPSCs with the PI3K inhibitor PX-866. This treatment restored phosphorylation of AKT (Ser^473^) and glycogen synthase kinase 3β (GSK3β) (Ser^9^) (Fig. S7A) and significantly increased the MES cell population to WT levels (Fig. S7B and C). MES marker expression was similarly rescued (Fig. S7D), indicating that *PTEN* regulates MES specification via its phosphatase activity.

To confirm this, we overexpressed a catalytically inactive *PTEN* mutant (D92A/C124A; PTEN-ΔCat) in *PTEN^−/−^* iPSCs. This mutant lacked phosphatase activity (Fig. 4B) [37] and failed to restore MES generation (Fig. 4C and D) or MES marker gene expression (Fig. 4E), reinforcing that PTEN’s phosphatase function is required for MES lineage induction.

*PTEN* has also been shown to promote collective cell migration in tumor models by suppressing AMP-activated protein kinase (AMPK) signaling [38]. During gastrulation, Epi cells undergo EMT and migrate through the PS to form mesoderm and DE [39]. Replated MES cells from *PTEN^−/−^* iPSCs exhibited markedly impaired convergence and collective migration, in contrast to WT cells (Fig. S6D). These findings underscored the critical role of *PTEN* in regulating this migratory behavior, indicative of the acquisition of mesenchymal characteristics. Notably, PTEN-ΔCat failed to rescue migration (Fig. 4F), and phosphoproteomic analysis identified enrichment of migration-related tight junction pathways (Fig. 4G). These results are consistent with previous reports that PTEN plays roles in stabilizing intercellular junctions by regulating PI3K/AKT signaling [40,41]. The long isoform of PTEN, PTEN-L, is a membrane-permeable lipid phosphatase that is secreted from cells and can be taken up by neighboring cells, and PTEN-L opens a new avenue to restoring PTEN function, particularly in tumors showing complete loss of *PTEN* [36]*.*

Interestingly, *PTEN* loss also induced prominent nuclear fragmentation and promoted redistribution of *SOX17* and *EOMES* proteins from the nucleus to the cytoplasm (Fig. S6D), a phenotype similarly observed in *PTEN^−/−^* DE cells (Fig. S3G). These findings align with the role of nuclear *PTEN* in preserving chromosomal integrity [33] and implicate nuclear-localized *PTEN* in the regulation of MES lineage fidelity.

### Multi-omic profiling identifies RA signaling as a PTEN-regulated pathway in mesendodermal differentiation

**Fig. 5.**
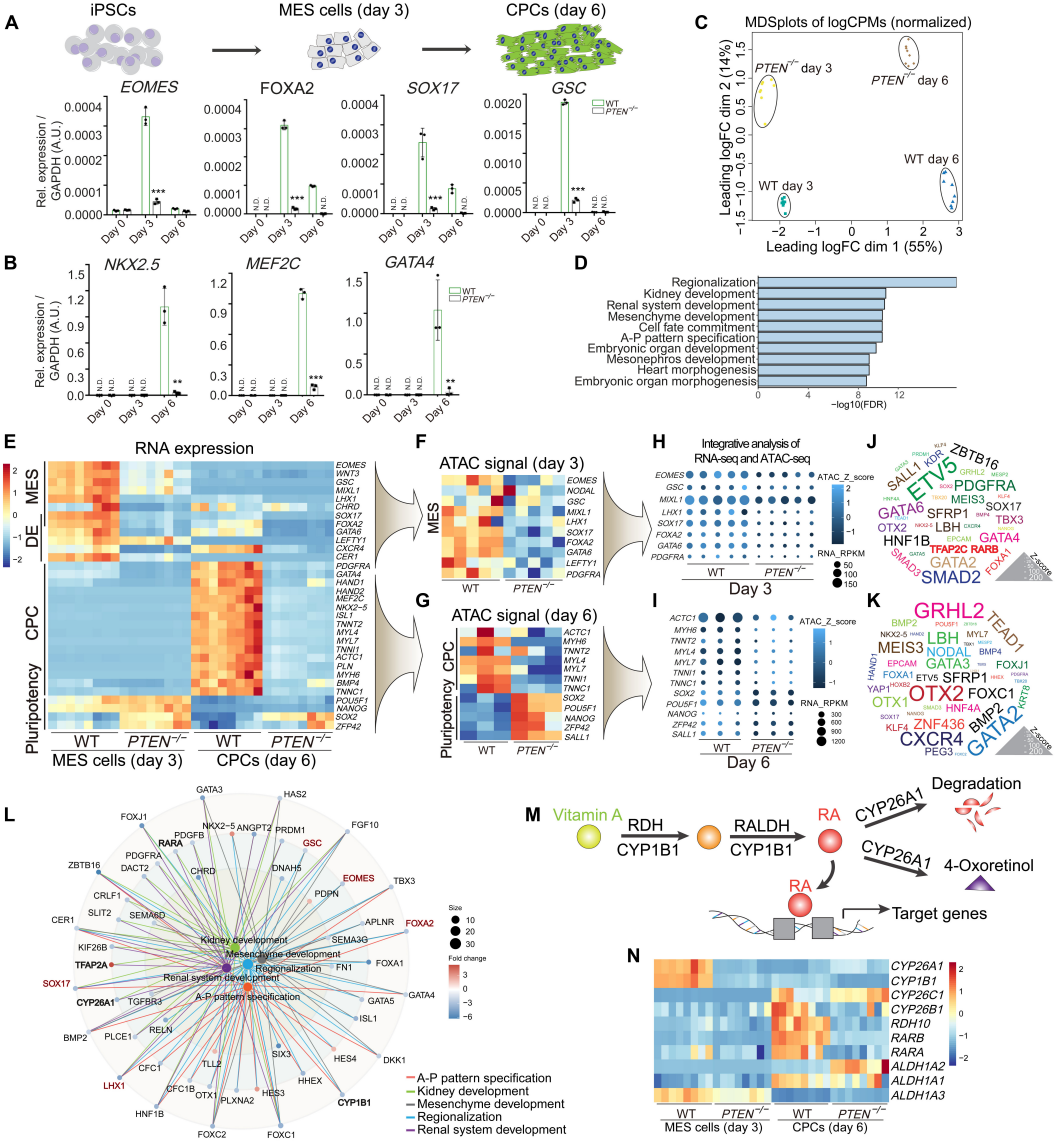
Multi-omic analyses identify RA signaling regulators in MES cell generation. (A and B) mRNA expression of MES and CPC markers at days 3 and 6, respectively, showing that *PTEN* ablation disrupts their temporal induction (*n* = 3, **P < 0.01; ***P < 0.001). (C) PCA of transcriptomic data separates WT and *PTEN^−/−^* samples at both differentiation stages. (D) Gene Ontology (GO) enrichment analysis of RNA-seq data highlights pathways involved in mesodermal and endodermal lineage specification. (E) Heatmap showing expression of MES, DE, CPC, and pluripotency markers in WT versus *PTEN^−/−^* MES and CPC populations. (F) Heatmap illustrating chromatin accessibility of MES genes at day 3 in WT and *PTEN^−/−^* MES cells. (G) Heatmap depicting chromatin accessibility of CPC and pluripotency genes at day 6 in WT and *PTEN^−/−^* CPCs. (H and I) Integrative analysis of ATAC-seq and RNA-seq data for MES genes at day 3, and for CPC and pluripotency genes at day 6. (J and K) Word cloud analysis of ATAC-seq data revealed motif enrichment in WT MES and CPC cells. (L) Pathway network analysis revealing significant enrichment of key RA signaling regulators in WT compared to *PTEN^−/−^* MES cells. (M and N) Heatmaps indicate that CYP26A1, a key RA catabolic enzyme, is significantly down-regulated in *PTEN^−/−^* MES cells.

To investigate *PTEN*’s role in MES differentiation, we induced CPC differentiation from MES cells and analyzed samples at days 3 and 6. At day 3, *PTEN^−/−^* cells exhibited significantly reduced MES marker expression compared to WT, with both groups showing diminished MES marker levels at day 6 (Fig. 5A). However, CPC marker expression was markedly lower in *PTEN^−/−^* cells at day 6, with no detectable MES gene expression (Fig. 5B). Transcriptional profiling across differentiation stages, performed in 2 independent batches, revealed distinct gene expression trajectories between WT and *PTEN^−/−^* groups. Batch effects were evident, particularly in the WT CPCs (Fig. S6E). To address this, batch correction was applied to raw count matrices using batch effect adjustment for RNA-seq count data (ComBat-seq). Multidimensional scaling (MDS) analysis following correction demonstrated clear segregation by genotype and differentiation time point (Fig. 5C), and pathway enrichment analysis identified significant down-regulation of signaling pathways involved in meso-endodermal organogenesis and anterior–posterior (A–P) patterning in *PTEN^−/−^* cells at day 3 (Fig. 5D), consistent with *PTEN*’s role in early lineage commitment from iPSCs.

Chromatin accessibility profiling using assay for transposase-accessible chromatin using sequencing (ATAC-seq) revealed that *PTEN* deletion markedly reduced accessibility at the loci of MES-specific genes, such as *SOX17* and *FOXA2*, at day 3, and CPC markers, including *MYL7* and *MYL4*, at day 6 (Fig. S6F). Complementary transcriptomic analysis showed significant down-regulation of MES and DE markers in *PTEN^−/−^* cells at day 3, followed by a decline in cardiac lineage gene expression and a concurrent up-regulation of pluripotency-associated genes at day 6 (Fig. 5E). These results are consistent with previous findings implicating *PTEN* in both cardiac differentiation and pluripotency regulation [34,42]. The ATAC-seq data further supported these transcriptomic trends, demonstrating reduced chromatin accessibility at MES and cardiac loci in *PTEN^−/−^* cells at days 3 and 6, respectively, while chromatin accessibility at pluripotency-associated regions was increased (*SOX2*, *POU5F1*, *NANOG*, *ZFP42*, *SALL1*) (Fig. 5F and G). A bubble plot integrating ATAC-seq and RNA-seq data further substantiated these findings, showing that *PTEN* ablation significantly diminished accessibility at MES gene loci at day 3 and CPC gene loci on day 6, while enhancing accessibility at loci associated with pluripotency (Fig. 5H and I).

Motif enrichment analysis revealed that WT MES cells preferentially enriched for transcription factors (TFs) critical to gastrulation, including *ETV5*, *SMAD2*, *GATA2*, *SALL1*, *PDGFRA*, *SFRP1*, *SOX17*, *HNF1B*, *GATA4*, and *GATA6* (Fig. 5J). Additionally, cardiac-related TFs such as *HAND1*, *NKX2.5*, *LBH*, *TEAD1*, and *MEF2C* were significantly enriched in WT cells relative to *PTEN^−/−^* CPCs at day 6 (Fig. 5K). These results collectively indicate that *PTEN* is essential for maintaining chromatin accessibility at MES and CPC gene loci, aligning with the transcriptomic data (Fig. 5E to K).

Pathway network analysis of transcriptome data revealed significant enrichment in mesodermal and endodermal lineage development pathways (Fig. 5L). MES-related TFs, including *LHX1*, *SOX17*, *FOXA2*, *EOMES*, and *GSC*, were also markedly up-regulated in WT MES cells (Fig. 5L). In addition to developmental regulators, pathway network analysis identified key components of the RA signaling cascade—*RARA*, *CYP26A1*, *CYP1B1*, and *TFAP2A*—as significantly enriched in WT compared to *PTEN^−/−^* MES cells (Fig. 5L). Vitamin A, an essential micronutrient during pregnancy, exerts broad developmental effects but is teratogenic in excess, especially during the first 60 d post-conception [43]. Its bioactive metabolite, RA, regulates early embryonic patterning by instructing the posterior neuroectoderm and foregut endoderm while permitting trunk mesoderm development [44]. Heatmap visualization of RA pathway regulators revealed robust expression of *CYP26A1* in WT MES cells, whereas no significant difference was observed between WT and *PTEN^−/−^* CPCs, indicating *CYP26A1*’s distinct roles in MES specification (Fig. 5M and N). *CYP26A1* is essential for spatially restricting RA signaling during embryogenesis [45]. scRNA-seq analysis revealed specific *CYP26A1* expression in the MES-1 cluster (Fig. 2E), which represents the population most similar to APS cells. Collectively, these findings indicate that *CYP26A1*-mediated RA signaling acts as a critical downstream effector of *PTEN* in regulating MES cell generation.

### CYP26A1 functions downstream of PTEN in MES specification

**Fig. 6.**
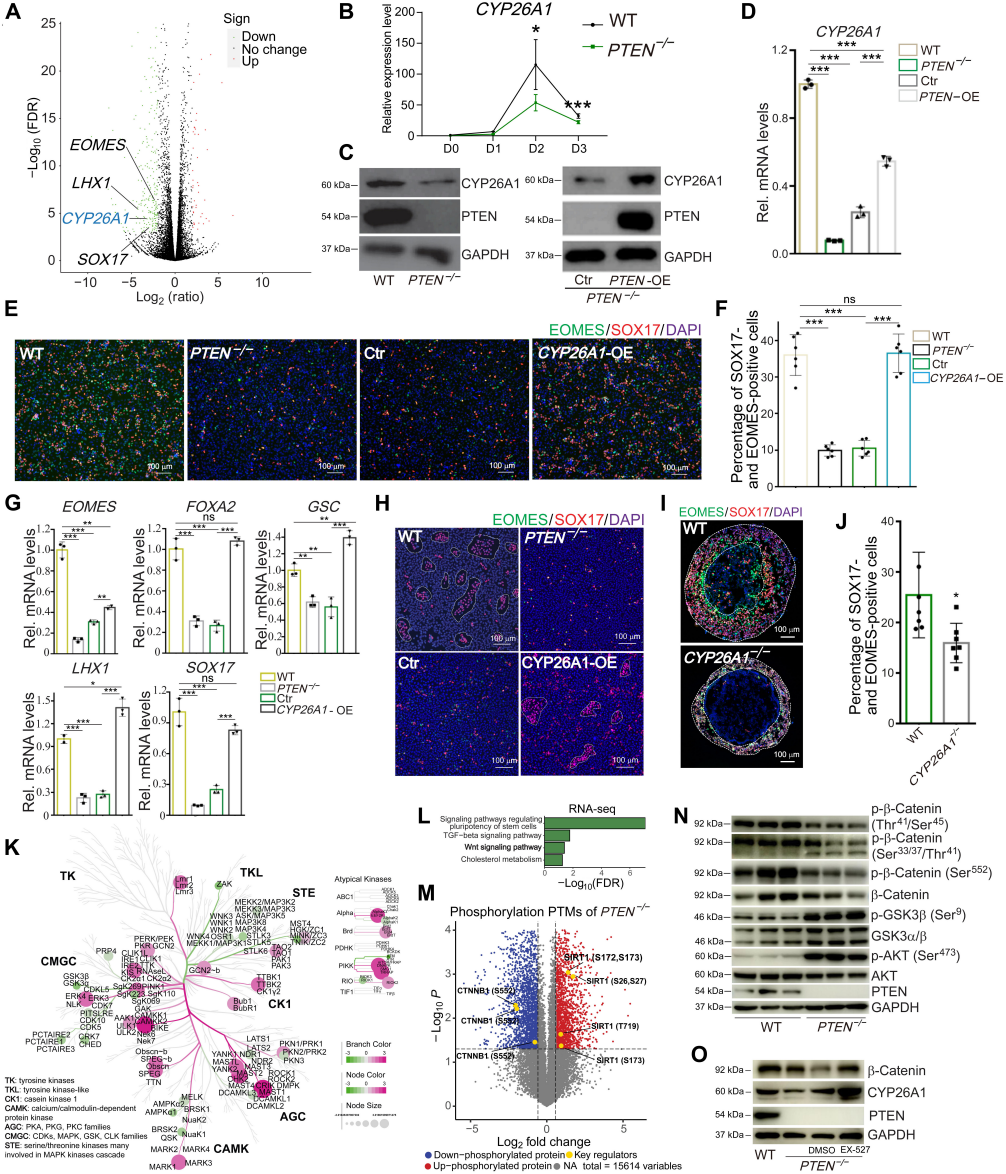
PTEN regulates CYP26A1 expression during MES lineage specification. (A) Volcano plot showing differentially expressed genes between WT and *PTEN^−/−^* MES cells, and MES markers are notably down-regulated in the *PTEN^−/−^* group. (B) Dynamic changes in the expression of *CYP26A1* between WT and *PTEN^−/−^* organoids (*n* = 3, *P < 0.05; ***P < 0.001). (C and D) *PTEN*-OE restores expression at both transcript and protein levels of *CYP26A1* (*n* = 3, ***P < 0.001). (E and F) *CYP26A1*-OE in *PTEN^−/−^* iPSCs rescues MES cell generation, as evidenced by restored coexpression of SOX17 and EOMES. Scale bar, 100 μm (*n* = 6, ***P < 0.001). (G) The expression levels of MES markers are rescued following *CYP26A1*-OE in *PTEN^−/−^* cells (*n* = 3, *P < 0.05; ***P < 0.01; ***P < 0.001). (H) *CYP26A1*-OE fully rescues MES cell migration impaired by *PTEN* loss. Scale bar, 100 μm. (I) *CYP26A1^−/−^* MES organoids show a marked reduction in SOX17- and EOMES-positive MES cells. Scale bar, 100 μm. (J) Proportion of MES cells coexpressing SOX17 and EOMES in WT and *CYP26A1^−/−^* MES organoids (*n* = 6, *P < 0.05). (K) Kinome dendrogram derived from human phosphorylation proteomics data illustrating kinase signaling pathways enriched in WT and *PTEN^−/−^* MES cells. (L) WNT signaling pathways are significantly enriched in the WT MES transcriptome. (M) Phosphoproteomic profiling reveals reduced phosphorylation of β-catenin (Ser^552^) and increased phosphorylation of SIRT1 in *PTEN^−/−^* cells. (N) Western blot analysis shows increased AKT and GSK3β phosphorylation and decreased β-catenin phosphorylation in *PTEN^−/−^* cells. (O) Treatment with SIRT1 inhibitor EX-527 restored the expression of β-catenin and CYP26A1 in *PTEN^−/−^* cells.

A volcano plot analysis of transcriptomic data revealed that *CYP26A1*, along with multiple MES marker genes, was significantly down-regulated in *PTEN^−/−^* MES cells (Fig. 6A). We examined the temporal expression dynamics of *CYP26A1* during MES organoid formation in WT and *PTEN^−/−^* groups. *CYP26A1* expression was markedly decreased in *PTEN^−/−^* MES organoids at day 2 (Fig. 6B), consistent with the decreased MES gene expression (Fig. 3D). Western blotting confirmed a marked reduction in *CYP26A1* protein levels following *PTEN* loss (Fig. 6C). In comparison, *PTEN*-OE restored *CYP26A1* expression at both the mRNA and protein levels (Fig. 6C and D), establishing *CYP26A1* as a downstream effector of *PTEN*. Importantly, overexpression of *CYP26A1* (*CYP26A1*-OE) in *PTEN^−/−^* iPSCs fully rescued the population of MES cells coexpressing *SOX17* and *EOMES* (Fig. 6E and F), and reinstated the expression of MES markers suppressed by *PTEN* deletion (Fig. 6G). Moreover, impaired MES cell migration induced by *PTEN* loss was completely rescued by *CYP26A1*-OE, restoring migration to WT levels (Fig. 6H). These findings support previous studies showing that *PTEN* is essential for Epi migration during mouse gastrulation [23].

Conversely, *CYP26A1* knockdown via short hairpin RNA (shRNA) significantly impaired MES cell generation and down-regulated MES markers (Fig. S8A to C). Additionally, we generated *CYP26A1^−/−^* iPSCs and observed that the ablation of *CYP26A1* significantly inhibited MES formation by reducing the proportion of MES cells as well as the mRNA levels of MES marker genes (Fig. S8D to F). As anticipated, MES organoids derived from *CYP26A1^−/−^* iPSCs also demonstrated a substantial decrease in *SOX17*- and *EOMES*-positive cells (Fig. 6I and J), which is fully consistent with the findings from *PTEN^−/−^* iPSCs (Fig. 3A and B). A kinome dendrogram from phosphoproteomic analysis revealed widespread alterations in kinase activity following *PTEN* deletion, particularly the WNT/GSK3β-related signalings (GSK3β, GSK3α, MARK1, MARK2, MARK3, MARK4, AMPKα1, AMPKα2, WNK1, WNK2, WNK3, WNK4, MEKK1/MAP3K1, MEKK2/MAP3K2, and MEKK3/MAP3K3) (Fig. 6K). Transcriptome data analyses also indicated the enriched WNT signaling pathway (Fig. 6L).

Phosphoproteomic profiling demonstrated significant suppression of β-catenin signaling in *PTEN^−/−^* cells (Fig. 6M), consistent with the role of stabilized β-catenin in inducing *CYP26A1* expression [46]. Furthermore, treatment of *PTEN^−/−^* cells with the PI3K inhibitor PX-866 elevated total and phosphorylated β-catenin (Ser^552^) and restored *CYP26A1* expression (Fig. S9A). WNT/β-catenin signaling pathway was further validated by Western blot analysis that *PTEN* deletion significantly suppressed the WNT/β-catenin signalings (Fig. 6N). Additionally, both PX-866 and *PTEN*-OE, but not catalytically inactive PTEN (PTEN-ΔCat), rescued MES marker expression (Fig. S9B and C). Heatmap analysis and kinome profiling further confirmed the up-regulation of WNT signaling regulators (Fig. S9D and E). These data suggest that *PTEN* drives MES specification via *CYP26A1*, acting through phosphatase-dependent mechanisms that converge on RA and WNT signaling pathways.

Notably, *PTEN^−/−^* MES cells also exhibited elevated phosphorylation of SIRT1, a nicotinamide adenine dinucleotide-dependent deacetylase, at S26, S27, S172, and S173—modifications associated with increased deacetylase activity (Fig. 6M). Western blot analysis confirmed the increased SIRT1 phosphorylation in the *PTEN^−/−^* MES cells (Fig. S9F). We further observed that treatment with the SIRT1 inhibitor EX-527 significantly restored the protein levels of both CYP26A1 and β-catenin, thereby providing additional evidence supporting the regulatory role of SIRT1 in the β-catenin/CYP26A1 signaling pathway (Fig. 6O). Moreover, we observed a significant increase of MES induction by EX-527 treatment in the *PTEN^−/−^* group, further confirming SIRT1’s involvement in PTEN-regulated MES generation (Fig. S9G and H). Given *SIRT1*’s role in regulating stem cell fate through β-catenin deacetylation [47,48], these findings suggest that enhanced SIRT1 activity in *PTEN^−/−^* cells may destabilize β-catenin via increased deacetylation, further disrupting *CYP26A1* transcription (Fig. S9I).

### *PTEN* facilitates MES specification by regulating RA homeostasis

RA is synthesized in distinct embryonic regions and regulates transcription by binding nuclear RA receptors (RARs), which in turn interact with RA response elements (RAREs) near target genes. However, excessive RA is known to be teratogenic [49]. *CYP26A1*, an RA-inducible cytochrome P450 enzyme, degrades RA into polar metabolites such as 4-hydroxy-RA and 4-oxoretinol (4-oxo-RA) [50]. *CYP26A1* plays a critical role in maintaining RA homeostasis by depleting excessive RA within specific embryonic regions, thereby shaping the A–P axis.

**Fig. 7.**
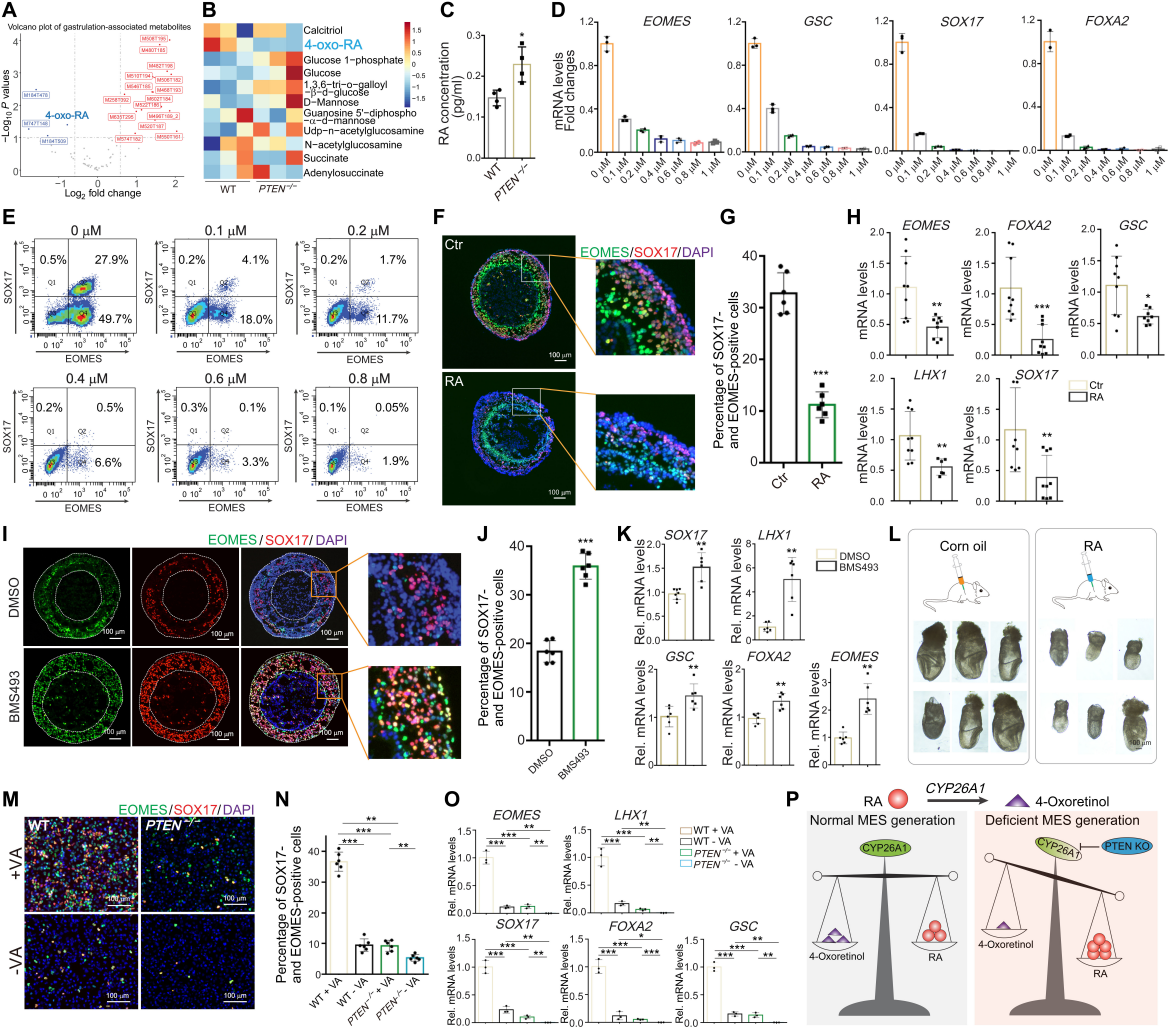
Both excessive and insufficient RA impairs MES differentiation. (A) Dot plot of metabolomics data shows significant reduction of 4-oxo-RA in *PTEN^−/−^* MES cells. (B) Heatmap profiling of gastrulation-related metabolites confirms marked depletion of 4-oxo-RA following *PTEN* ablation. (C) RA concentrations in WT and *PTEN^−/−^* MES cells. (D) qPCR analysis reveals dose-dependent suppression of MES marker genes in WT cells upon exogenous RA treatment. (E) Flow cytometric analysis shows reduced MES cell proportions following RA exposure. (F and G) RA treatment reduced SOX17- and EOMES-positive cells in MES organoids. Scale bar, 100 μm (*n* = 6, ***P < 0.001). (H) qPCR confirms down-regulation of MES markers in RA-treated MES organoids (*n* = 9, *P < 0.05; **P < 0.01; ***P < 0.001). (I and J) MES organoid assays confirm that RA antagonist BMS493 enhances MES formation. Scale bar, 100 μm (*n* = 6, ***P < 0.001). (K) qPCR data show robust up-regulation of MES markers following RA inhibition (*n* = 6, **P < 0.01). (L) Morphological comparison of control and RA-treated embryos illustrates developmental delays with excess RA. Scale bar, 100 μm. (M and N) IF staining of MES cell formation under vitamin A-supplemented versus vitamin A-depleted conditions. Scale bar, 100 μm (*n* = 6, **P < 0.01; ***P < 0.001). (O) qPCR analysis demonstrates that both vitamin A and RA are essential, but tightly regulated, for proper MES cell differentiation (*n* = 3, *P < 0.05; **P < 0.01; ***P < 0.001). (P) Schematic model proposing that CYP26A1-mediated RA degradation is critical for maintaining RA homeostasis during MES specification.

To examine whether RA participates in *PTEN*’s effect on MES generation, we performed lipid metabolite profiling focused on gastrulation-associated metabolites. Notably, 4-oxo-RA levels were significantly decreased in *PTEN^−/−^* MES cells (Fig. 7A), a finding confirmed by heatmap analysis showing diminished 4-oxo-RA upon *PTEN* deletion (Fig. 7B). Enzyme-linked immunosorbent assay (ELISA) also revealed elevated RA levels in *PTEN^−/−^* MES cells (Fig. 7C). These results suggest that loss of *PTEN* reduces *CYP26A1* expression, impairing RA catabolism and resulting in RA accumulation in *PTEN^−/−^* MES cells.

We hypothesized that elevated RA levels suppress MES differentiation in *PTEN*-deficient cells. To test this, WT iPSCs were treated with 1 μM RA, resulting in a significant reduction in the expression of MES markers, closely recapitulating the expression profile observed in *PTEN^−/−^* MES cells (Fig. S10A). RA exposure led to a dose-dependent inhibition of MES differentiation (Fig. S10B), with progressive down-regulation of MES markers including *EOMES*, *FOXA2*, *SOX17*, and *GSC*, mirroring the expression profile observed in *PTEN^−/−^* MES cells (Fig. 7D). Flow cytometric analysis confirmed reduced MES cell proportions following RA treatment (Fig. 7E). Similarly, in MES organoids, RA treatment decreased MES cell populations and marker expression (Fig. 7F to H and Fig. S10C).

To confirm the inhibitory role of RA in MES specification, we treated *PTEN^−/−^* iPSCs with the pan-RAR antagonist BMS493. BMS493 significantly increased MES cell proportions (Fig. S10D) and partially restored MES gene expression (Fig. S10E). In *PTEN^−/−^* MES organoids, BMS493 treatment similarly enhanced the number of SOX17- and EOMES-positive cells as well as the expression of MES markers (Fig. 7I to K and Fig. S10F). In vivo, pregnant mice treated with RA at E4 yielded E7.5 embryos that were smaller (Fig. 7L), phenocopying *PTEN^−/−^* embryonic phenotypes (Fig. 3E and F).

RA signaling is essential for the development of multiple organ systems, including the axial skeleton, spinal cord, heart, and reproductive tract [51]. Recent studies also show that vitamin A, the RA precursor, restricts lineage plasticity and guides stem cell fate decisions [52], and that RA induces posterior embryonic structures in human gastruloids [53]. Consistently, withdrawal of vitamin A from the MES differentiation medium markedly suppressed MES generation in both WT and *PTEN^−/−^* cells, as evidenced by reduced numbers of SOX17- and EOMES-positive cells and decreased expression of MES markers (Fig. 7M to O), underscoring the essential role of RA in MES lineage specification. Collectively, these data indicate that both RA excess and deficiency impair MES differentiation, and that *CYP26A1* maintains RA homeostasis critical for proper embryonic patterning (Fig. 7P).

## Discussion

Specification of MES at the PS is a transient yet critical developmental milestone. Due to the inaccessibility and complexity of early human embryos, our understanding of native human PS formation remains limited, highlighting the need for sophisticated in vitro models. Here, we described an improved organoid system based on a previously published protocol [25]. Comprehensive characterization revealed that these MES organoids closely resemble the cellular composition of the APS, rather than the PPS. The MES organoids faithfully recapitulate human MES lineage specification and can further differentiate into EC and DE organoids (Fig. 1), which may serve as progenitors for vascular, hepatic, or pancreatic tissues. Collectively, this system provides a valuable platform for dissecting early lineage specification, modeling congenital disorders, and facilitating the discovery of drugs targeting developmental abnormalities.

The incidence of spontaneous abortion ranges from 10% to 15%, with many cases linked to genetic anomalies that may contribute to congenital growth and developmental disorders [54]. *PTEN* loss-of-function mutations are known to cause embryonic lethality, and aberrant *PTEN* expression has been associated with spontaneous abortion in clinical settings [20–22]. RA signaling plays a crucial role in embryogenesis, including cell–cell communication and organ formation [44], but dysregulated RA levels—whether excessive or deficient—can impair early development, especially blastocyst formation and implantation [55]. Although RA is essential for early patterning, its role in PS formation, particularly in the specification of APS-derived lineages, remains unclear [44]. Our study revealed that *PTEN^−/−^* MES cells exhibit significantly reduced levels of 4-oxo-RA (Fig. 7A and B), a CYP26A1-catalyzed RA metabolite, suggesting elevated intracellular RA. These elevated RA levels suppress MES lineage specification (Fig. 7D to H and Fig. S10A to C). Since *Pten*-null embryos fail to survive beyond E7.5 [23], we propose that *PTEN* loss leads to RA imbalance, contributing to the observed developmental arrest. On the other hand, vitamin A or RA deficiency during pregnancy can cause fetal defects such as night blindness, skeletal malformations, and epithelial dysplasia [56]. Our results demonstrated that RA deficiency similarly disrupts MES differentiation (Fig. 7M to O), aligning with clinical observations of RA’s dose-sensitive teratogenicity [56,57]. These findings establish *PTEN* as a key regulator of RA homeostasis via *CYP26A1*, safeguarding APS lineage commitment.

Our data indicate that *PTEN* loss suppresses β-catenin protein levels and its phosphorylation, which may disrupt *CYP26A1* transcription (Fig. 6M and N). Notably, the phosphorylation sites affected (Thr^41^/Ser^45^/Ser^33^/Ser^37^) are linked to β-catenin degradation, suggesting a complex mechanism by which PTEN stabilizes β-catenin. *PTEN^−/−^* MES cells also exhibited elevated phosphorylation of SIRT1 (Fig. 6M and Fig. S9F). Particularly, inhibition of SIRT1 significantly increased the expression levels of CYP26A1 and β-catenin (Fig. 6O), as well as MES generation (Fig. S9G and H), indicating that SIRT1 participates in the PTEN/β-catenin/CYP26A1 signaling axis. These findings suggest that elevated SIRT1 activity in *PTEN^−/−^* cells may promote β-catenin destabilization through enhanced deacetylation. Consistent with this, we propose that *PTEN* loss leads to reduced β-catenin protein levels, decreased phosphorylation at Ser^552^, and increased phosphorylation of SIRT1 at Ser^27^. These effects are likely mediated by SIRT1-dependent destabilization of β-catenin, thereby further impairing CYP26A1 transcription (Fig. S9I).

This model thus serves as a robust platform for studying lineage commitment during early human development. This work identifies *CYP26A1*-mediated RA signaling as a central node in *PTEN*’s function, providing novel insights into the regulatory networks potentially driving human gastrulation and PS formation. Deciphering the molecular mechanisms by which *PTEN* orchestrates MES specification and APS formation enhances our understanding of the pathogenesis underlying certain congenital malformations and spontaneous miscarriages.

## Materials and Methods

### Cell culture, MES cell generation, and MES organoid formation

hiPSCs (Allen Institute for Cell Science; AG28869, GM23720, AICS-0060-027) were cultured using mTeSR1 medium (STEMCELL Technologies, 85850). For 2D MES cell generation, hiPSCs were seeded at a density of 250,000 cells per well in 12-well plate in mTeSR1+ROCKi (Y-27632, Selleckchem, S1049). After 48 h of culture, MES differentiation was initiated by replacing the medium with RPMI 1640 medium (Life Technologies, 11875085) supplemented with 2% B-27 without insulin (Life Technologies, A1895601) and 6 μM CHIR99021 (Selleckchem, S1263). Following 48 h of induction, the medium was replaced with RPMI 1640 medium containing 2% B-27 supplement without insulin for 24 h. Cells were then harvested for qPCR, Western blot, and IF staining.

For MES organoid formation, we modified the protocol from Drakhlis et al. [25]. hiPSCs (Allen Institute for Cell Science; AG28869, GM23720, AICS-0060-027) at passage 8 were seeded at 5,000 cells per well in a U-shaped ultralow-attachment 96-well plate (Corning, 3788) in mTeSR1+ROCKi. The plates were centrifuged at 300*g* and incubated to facilitate aggregate formation. After 48 h, the cell aggregates were embedded in Matrigel droplets (Corning, 354234). Differentiation was initiated on day 0 by replacing the medium with RPMI 1640 medium containing 2% B-27 supplement without insulin and 7.5 μM CHIR99021. After 24 h, the medium was replaced with RPMI 1640 medium containing 2% B-27 without insulin. Following an additional 48 h, MES organoids were collected for downstream analyses.

### DE cell generation from hiPSCs

hiPSCs at passage 8 were seeded at 250,000 cells per well in 12-well plates in mTeSR1+ROCKi. After 48 h of culture, MES differentiation was initiated by replacing the medium with RPMI 1640 medium supplemented with 2% B-27 without insulin, 3 μM CHIR99021, and 100 ng/ml activin A (R&D Systems, 338-AC-050). After 24 h, the medium was replaced with RPMI 1640 medium containing 2% B-27 supplement and 100 ng/ml activin A for an additional 2 d. Subsequently, DE cells were harvested for qPCR, flow cytometric analysis, and IF staining.

### DE and EC organoid formation

After collection of the MES organoids, differentiation of DE and EC organoids was initiated. For DE organoid generation, the culture medium was replaced with RPMI 1640 medium supplemented with 2% B-27 without insulin and 100 ng/ml activin A for an additional 3 d. For EC organoid generation, MES organoids were cultured in RPMI 1640 medium containing 2% B-27 without insulin, 100 ng/ml VEGF (PeproTech, 100-20), and 2 μM forskolin (Selleckchem, S2449) for 48 h. Subsequently, the medium was exchanged to RPMI 1640 medium containing 2% B-27 supplement, 100 ng/ml VEGF, 100 ng/ml fibroblast growth factor-2 (FGF-2) (PeproTech, AF-100-18B), and 15% fetal bovine serum for an additional 5 d.

### Laboratory animals

*PTEN^+/−^* C57BL/6 mice at 2 months of age were intercrossed to generate WT and *PTEN^−/−^* embryos. Embryos were harvested at E7.5 for genotyping and IF staining, and all animal experiments were conducted in accordance with the guidelines and regulations of Shandong University. To minimize suffering, anesthesia was induced by intraperitoneal ketamine (100 mg/kg)/xylazine (10 mg/kg), maintained with 1.5% to 2% isoflurane, and analgesia provided via buprenorphine (0.1 mg/kg subcutaneous) pre-/post-procedure. Euthanasia was performed by gradual CO₂ inhalation (30% to 70% chamber volume/min displacement rate) followed by cervical dislocation or bilateral thoracotomy to ensure death, per AVMA Tier I methods; tissues were rapidly collected into ice-cold phosphate-buffered saline (PBS) or 4% paraformaldehyde (PFA) for downstream analyses. The experimental protocol was approved by the Animal Care and Research Committee of Shandong University.

### Genome-edited iPSC generation

*PTEN* and *CYP26A1* deletion in human iPSC were generated as previously detailed [34,58]. *PTEN^−/−^* iPSCs were derived from 2 different iPSC lines (Allen Institute for Cell Science; AG28869, AICS-0060-027) and *CYP26A1^−/−^* iPSCs from iPSC lines (Allen Institute for Cell Science; AG28869). Guide RNA sequences were incorporated into the pSpCas9 (BB)-2A-GFP vector (Addgene, 48138), which encodes both Cas9 nuclease and green fluorescent protein (GFP). iPSCs were cultured in 6-well plates and transfected with the Cas9-single-guide RNA constructs using Lipofectamine 3000 (Invitrogen). Two days post-transfection, GFP-positive cells were isolated by fluorescence-activated cell sorting (FACS). The enriched, gene-edited cell populations were subsequently cultured in 48-well plates for 4 to 5 d, followed by dilution and plating into 96-well plates to enable single-colony formation. Individual colonies were expanded and screened for *PTEN^−/−^* and *CYP26A1^−/−^* genotypes via DNA sequencing. Primer sequences used for *CYP26A1* deletion are provided in Table S1.

### Plasmid construction

Human *CYP26A1* genes were amplified by RT-PCR from total RNA isolated from hiPSCs and subsequently cloned into the pCDH-EF1-MCS-BGH-PGKcopGFP-T2A-Puro Vector (System Biosciences, CD550A-1). The primer sequences are provided in Table S1. The *PTEN*-OE and *PTEN*-ΔCat-OE system were previously described [34, 37]. To achieve knockdown of *CYP26A1* expression, shRNA constructs were generated and cloned into pLKO.1 vector. The shRNA oligonucleotide sequences are listed in Table S1.

### Cryosectioning and staining

Organoids were harvested and subsequently fixed in 4% PFA. Following fixation, samples were permeabilized with 0.3% Triton X-100 for 20 min. Following washing with PBS, organoids were stored at 4 °C until further processing. For cryo-embedding, organoids were transferred into tissue embedding molds containing optimal cutting temperature compound and rapidly frozen at low temperatures. Tissue sections of 10-μm thickness were then prepared using either the Epredia Cryostar NX70 Cryostat or the Leica CM1950 Cryostat.

### Immunofluorescent staining

The cells were fixed in 4% PFA for 20 min at room temperature, followed by 3 washes with PBS for 5 min each. Subsequently, the samples were incubated with permeabilization buffer and washed 3 additional times with PBS. To minimize nonspecific binding, the samples were blocked with a blocking buffer for 20 min at room temperature. Thereafter, the cells were incubated overnight at 4 °C, with primary antibodies diluted in staining buffer. The following day, samples were incubated with secondary antibodies for 1 h at room temperature in the dark. Finally, fluorescence imaging was performed using a fluorescence microscope. Primary antibodies were applied at the following dilutions: anti-SOX17 (R&D Systems, AF1924, 1:1,000), anti-EOMES (Cell Signaling Technology, 81493, 1:1,000), anti-FoxA2 (Cell Signaling Technology, 8186, 1:1,000), and anti-CDH5 (Cell Signaling Technology, 2500, 1:1,000).

### Flow cytometric analysis

Both 2D cultured cells and 3D organoids were collected into tubes and digested into single-cell suspensions using Accutase (Sigma, A6964) for 10 min at 37 °C. The digested cells were fixed with 4% PFA for 10 to 15 min. Following fixation, cells were washed with 500 μl of FACS buffer and centrifuged at 1,500 rpm for 5 min at 4 °C. The supernatant was carefully aspirated. Cells were then permeabilized by incubation with 200 μl of 0.3% Triton-X100 buffer at room temperature for 15 min. After an additional wash with 500 μl of FACS buffer and centrifugation at 2,000 rpm for 5 min at 4 °C, the supernatant was discarded gently. Subsequently, cells were incubated on ice with primary antibodies (Anti-SOX17 antibody, anti-EOMES antibody; Anti-SOX17 antibody, anti-FoxA2 antibody) for 45 to 60 min. Following primary antibody staining, cells were washed with 500 μl of FACS buffer and centrifuged under the same conditions. Cells were then resuspended in 100 μl of secondary antibodies, mixed thoroughly, and incubated at room temperature for 20 to 30 min in the dark. After a final wash and centrifugation, cells were resuspended in FACS buffer and filtered with Falcon 5-ml round bottom polystyrene test tube to prepare for flow cytometry analysis.

### Western blot

Protein samples were prepared by lysing cells in radioimmunoprecipitation assay (RIPA) buffer supplemented with a protease-inhibitor cocktail for 20 min on ice. Lysates were then centrifuged at 12,000 rpm for 5 min at 4 °C, and the supernatant was collected. The protein extracts were mixed with 5× sodium dodecyl sulfate (SDS) sample buffer and denatured by heating at 98 °C for 10 min. Samples were loaded onto SDS–polyacrylamide gel electrophoresis gels and electrophoresed initially at 80 V, followed by 120 V until the dye front reached the bottom of the gel.

Following electrophoresis, polyvinylidene difluoride membranes were equilibrated by soaking in transfer buffer for 1 to 2 min and briefly rinsed. The gel and membrane were assembled in a transfer apparatus, and proteins were transferred at 110 V for 75 min. Subsequently, membranes were blocked in blocking buffer for 1 h at room temperature.

Primary antibodies diluted in blocking buffer were incubated with the membranes overnight at 4 °C. After incubation, membranes were washed 3 times with tris-buffered saline containing Tween 20 (TBST) for 10 min each. Membranes were then incubated with secondary antibodies diluted in blocking buffer for 1 h at room temperature, followed by 3 additional washes with TBST for 10 min each. The membranes were then ready for imaging and further analysis.

### Real-time PCR analysis

Total RNA was extracted from collected cells using TRIzol reagent (Invitrogen) according to the manufacturer’s instructions. Approximately 400 ng of RNA was reverse-transcribed into complementary DNA (cDNA). Quantitative PCR (qPCR) was performed using SYBR Green chemistry, and relative mRNA expression levels were calculated using the ΔΔCt method. Gapdh was selected as the endogenous reference gene for normalization. All qPCR assays were conducted with technical replicates to ensure reproducibility. Primer sequences are provided in Table S1.

### Determination of RA concentrations

RA concentrations were measured using a commercially available ELISA kit (MyBioSource, catalog no. MBS705877). The assay detection range was 0.625 to 10 ng/ml. Briefly, WT and *PTEN^−/−^* MES cells were lysed in RIPA buffer (Thermo Scientific, catalog no. 89901) and centrifuged at 10,000 rpm for 10 min at 4 °C. The resulting supernatants were collected and used for RA quantification according to the manufacturer’s instructions.

### Image processing and data quantification

IF staining images were analyzed using custom scripts in ImageJ. Cell counting was performed utilizing the StarDist plugin for ImageJ. 3D reconstruction and volumetric analysis of MES and EC organoids were conducted using the 3D ImageJ Suite.

### RNA velocity analysis

To infer differentiation trajectories of MES, CPCs, and DE cell clusters, we employed velocyto (v0.17.17) to quantify spliced and unspliced RNA reads using default parameters. Subsequent analyses and visualizations were performed with the Python packages scVelo (v0.2.4) and Dynamo (v1.1.0) for each dataset. Marker gene expression across different cell clusters was visualized using violin plots generated by the VlnPlot function from the Seurat package. These violin plots illustrate the distribution of marker gene expression levels within the respective cell populations.

### Bulk RNA-seq library prep

For bulk RNA-seq libraries, we extracted RNA from the WT and *PTEN^−/−^* MES cells and CPC cells using TRIzol (Invitrogen) according to the manufacturer’s protocol. Bulk RNA-seq was performed in 2 separate batches, with 3 biological replicates in the first batch and 5 biological replicates in the second batch. Bulk RNA-seq libraries were prepared by Ouyi Biomedical Technology Co. Ltd. (China) using the NEBNext Ultra II RNA Library Prep Kit (New England Biolabs, E7805S). The resulting libraries exhibited fragment sizes ranging from 220 to 500 base pairs (bp), with a peak at approximately 350 bp. Sequencing was performed on an BGISEQ-500 platform, generating 150-bp paired-end reads. For edgeR differential expression analysis, lowly expressed genes were first filtered out (raw counts < 50 per sample). Differentially expressed genes (DEGs) were then identified using the following criteria: expression percentage > 0.1, absolute log_2_ fold change > 2, and adjusted *P* value (Benjamini–Hochberg method) < 0.05. Volcano plots were generated using the “geom_point” function in the R package ggplot2. MDS plots were generated using normalized count data in edgeR to assess global transcriptomic similarity among samples. Distances were computed from the leading log_2_ fold changes of the most variable genes, and the first 2 dimensions were plotted to visualize clustering patterns and potential batch effects. Batch correction on the raw count matrices was performed using ComBat-seq from the sva R package.

### Bulk ATAC-seq

Bulk ATAC-seq libraries were prepared according to the ATAC-Seq Kit Manual (Active Motif, #53150). ATAC-seq was performed in 2 independent batches for WT and *PTEN*^*−/−*^ MES cells, comprising 3 biological replicates in the first batch and 5 biological replicates in the second batch. Briefly, 50,000 to 100,000 fresh cells were collected, and nuclei were extracted using ATAC lysis buffer. Subsequently, assembled Tn5 transposomes and 2× Tagmentation Buffer were added to the nuclei, followed by incubation to simultaneously fragment the DNA and insert sequencing adapters (tagmentation). The tagmented DNA was then purified using the provided DNA purification columns and buffers. PCR amplification was performed using Q5 High-Fidelity DNA Polymerase and indexed primers (i5 and i7) to generate Illumina-compatible libraries. Amplified libraries were cleaned with SPRI beads to remove primers and small fragments. Library size distribution was assessed using a fragment analyzer, with an expected size range of approximately 250 to 1,000 bp and a characteristic ~150-bp periodicity reflecting nucleosome spacing. Libraries were sequenced using paired-end Illumina sequencing, targeting a depth of at least 20 million to 30 million reads per sample.

### Read processing and quality control

Raw sequencing reads were assessed using FastQC. Adapter sequences and low-quality bases (quality score < 20) were trimmed using Cutadapt to generate clean reads. Reads shorter than 20 bp were excluded from downstream analysis.

### Read alignment and quality control

Clean reads were aligned to the reference genome using BWA. For ATAC-seq analysis, reads mapping to ENCODE blacklist regions and the mitochondrial genome were excluded. Low-quality alignments with a mapping quality score < 5 were filtered out. PCR duplicates were removed using Picard. To account for Tn5 insertion bias, aligned reads were shifted according to standard ATAC-seq conventions. Fragment sizes were subsequently classified into nucleosome-free regions (NFRs; <100 bp), mononucleosomes (180 to 247 bp), and dinucleosomes (315 to 473 bp). Signal profiles for NFR and mononucleosome fragments were generated, and enrichment of reads around transcription start sites (TSSs) was evaluated using ATACseqQC (R/Bioconductor).

### Peak calling and quality control

Peaks were identified using Genrich with default parameters. The fraction of reads in peaks (FRiP) was calculated as the proportion of mapped reads that overlap the called peak regions.

### Normalization and differential accessible analysis

Gene-level read counts were quantified using featureCounts. Raw count data were imported into DESeq2 (R/Bioconductor) for normalization and differential accessibility analysis. Counts were normalized using the reads per kilobase of transcript per million mapped reads (RPKM) method. Differentially accessible genes between WT and *PTEN^−/−^* samples were identified using a negative binomial generalized linear model implemented in DESeq2. Genes with an adjusted *P* value (Benjamini–Hochberg corrected) < 0.05 and absolute log_2_ fold change ≥ 1 were considered significantly differentially accessible. The ATAC-seq signal for each gene was quantified by summing the signal values of all peaks located within the gene. Peak signal values were calculated using Genrich. Bubble plots were generated to display both the ATAC-seq signal and RPKM values for selected transcription factors. The difference in ATAC-seq signal between day 3 and day 6 was calculated as the mean ATAC-seq value of samples collected at day 6 minus the mean value of samples collected at day 3.

### The word cloud figure method

The ATAC-seq signal for each gene was quantified by summing the peak signal intensities located within the gene region. Peak signal values were calculated using Genrich. The differential ATAC value between day 3 and day 6 was determined by subtracting the mean ATAC value at day 3 from the mean value at day 6 across samples. A word cloud was generated based on these data, where the frequency of transcription factors was replaced by their corresponding differential ATAC values between day 3 and day 6. The word cloud visualization was created using the “wordcloud2” R package, with larger characters representing transcription factors exhibiting greater differences in ATAC accessibility.

### ScRNA-seq for MES organoids

MES organoids were collected on day 3 and individually dissociated into single cells using Accutase. The dissociated cells were washed with PBS and centrifuged for 5 min; the cells were resuspended ensuring that there were above 100 × 10^3^ cells for each sample. Library preparation for scRNA-seq analysis of the mRNA was performed according to the Chromium NextGem Single Cell 3′ Reagent Kits v3.1 user guide. Briefly, a given excess of cells was loaded to the 10× controller to reach a target number of around 1 × 10^4^. Quantification of the libraries was performed using Qubit 4 Fluorometer.

ScRNA-seq was conducted using the NextSeq 2000 platform, following the manufacturer’s protocol. In brief, a single-cell suspension was loaded into the microfluidic chip together with gel beads, reverse transcription reagents, and oil. The microfluidic system partitioned the mixture into thousands to tens of thousands of Gel Beads-in-Emulsion (GEMs), each ideally encapsulating a single cell and a uniquely barcoded bead. Within each GEM, cells were lysed, and mRNA transcripts were captured by the bead-bound barcoded primers. Reverse transcription was subsequently performed inside the GEMs to synthesize complementary cDNA from the captured mRNA. Following reverse transcription, the GEMs were broken to release the barcoded cDNA, which was then amplified by PCR to generate sufficient material for downstream processing. The amplified cDNA was used to construct sequencing libraries compatible with Illumina sequencing platforms through the addition of sequencing adapters. Finally, the prepared libraries were sequenced on Illumina instruments, producing paired-end reads containing both cell barcodes and transcript sequences. The resulting RNA sequence data were aligned to the human reference genome for further analysis.

### ScRNA-seq raw data processing

The 10× Genomics Cell Ranger analysis pipeline (version 7.0.0) was employed using default parameters. Briefly, binary base call (BCL) files were demultiplexed into FASTQ files using the cellranger mkfastq command, incorporating the appropriate sample sheet and 10× Genomics-supplied barcodes. Subsequently, the cellranger count pipeline was used to align sequencing reads to the designated 10x Genomics reference genome, quantify reads aligning to each gene (including intronic reads in the count matrix), and perform initial clustering and generate summary statistics. Finally, the outputs from the cellranger count analyses for all samples were aggregated and normalized to equivalent sequencing depths, and feature-barcode matrices were recomputed using the cellranger aggr function.

### scRNA-seq analysis

The filtered RNA feature count matrix, comprising 36,601 features across 27,202 cells, was generated using Cell Ranger. A Seurat object was constructed using the Seurat R package after excluding features expressed in fewer than 3 cells, resulting in 27,924 features retained for downstream analysis.

Low-quality cells were filtered based on the following criteria: cells with fewer than 500 detected features (potential ambient RNA contamination; 170 cells removed), cells with more than 6,000 detected features (77 cells removed), and cells with mitochondrial gene content exceeding 20% (11 cells removed) (Fig. S2A). Doublet scores were calculated using Scrublet, and an automatically determined threshold of 0.47 was applied to identify doublets. Based on this threshold, 463 cells predicted as doublets were excluded from subsequent analyses (Fig. S2B).

The feature expression matrix was normalized and scaled using Seurat, followed by dimensionality reduction via principal components analysis (PCA). UMAP embeddings were computed using the top 50 principal components. A total of 15 cell clusters were identified. For each cluster, the top 10 significantly enriched marker genes were determined, and a heatmap was generated to visualize their expression patterns across clusters.

### Integrative analysis of scRNA-seq data from MES organoids and human and monkey embryos

Natural human and monkey embryo scRNA-seq datasets utilized for integration analysis were obtained from previously published studies [26,29]. Human MES organoid scRNA-seq data were projected onto the human and monkey embryo UMAP embedding to facilitate comparative analysis. Quality control and filtering of cells were performed using the Seurat v4.1.1 package, followed by data normalization. The scRNA-seq data for the human and monkey embryo were retrieved from the GSA-human database under accession number HRA005567 and the National Center for Biotechnology Information Gene Expression Omnibus (GEO) under accession number GSE193007 [26,27,29]. The provided count expression matrix and cell annotation files were used to construct a Seurat object with the Seurat (v4.1.1) R package. The human MES organoid and primate embryo Seurat objects were merged using the “merge” function and subsequently integrated via the “IntegrateLayers” function. The merged dataset was normalized using “NormalizeData” and scaled with “ScaleData”. UMAP embeddings were computed using the “RunUMAP” function. Cell annotations from the MES organoid Seurat clusters were appended to the merged object, labeling these cells accordingly, while primate embryo cells were annotated as “Human embryo” or “Monkey embryo”. Additionally, annotations from the reference dataset were incorporated, with MES organoid cells labeled as “APS organ”. Both annotations were visualized on the UMAP plots to distinguish cell populations (Fig. 2F and G).

A Sankey diagram was generated to visualize the distribution of MES organoid cells mapped to human and monkey embryo cell cluster (Fig. S2E and F). Seurat objects derived from human MES organoids and from human and monkey embryos were merged and integrated using the IntegrateLayers function in Seurat with the canonical correlation analysis (CCA) method. A 2D UMAP embedding was generated using the RunUMAP function based on the first 30 integrated CCA dimensions. For the human and monkey embryo dataset, cluster centroids were defined by averaging the UMAP coordinates of all cells within each cluster. Each human organoid cell was subsequently assigned to the nearest human and monkey embryo cluster based on Euclidean distance to these centroids. Cells with a minimum Euclidean distance greater than 5 were excluded from further analysis.

### Histological analysis

Mouse embryos were fixed overnight in 4% PFA, embedded in paraffin, and sectioned at a thickness of 5 μm. For immunohistochemistry, tissue sections were blocked for 30 min at room temperature, followed by incubation with primary antibodies overnight at 4 °C. The anti-FoxA2 antibody was employed for IF analyses.

### Phosphoproteomic analysis

In this study, each sample was independently prepared, with proteins enzymatically digested and phosphorylated peptides subsequently enriched prior to analysis by data-independent acquisition (DIA) mass spectrometry. The resulting DIA raw data files were imported into Spectronaut software for comprehensive analysis. For each sample, the numbers of identified phosphorylation sites, modified peptides, and phosphorylated proteins were quantified. To assess the distribution of phosphorylation sites across proteins, the total number of class I phosphorylation sites detected on all identified proteins was enumerated. Among the modified proteins, the average frequency of class I phosphorylation sites was calculated as 0.8 sites per 100 amino acids.

To identify differentially expressed phosphorylated proteins between experimental groups, the dataset was further filtered to retain statistically significant modifications. Recognizing that individual proteins may harbor multiple phosphorylation sites exhibiting distinct regulatory patterns, quantification was performed at the modified peptide level rather than at the protein level. Differentially modified peptides were defined using a fold-change threshold of ≥1.5 for up-regulation or ≤0.67 for down-regulation, combined with a significance cutoff of *P* < 0.05 (Student’s *t* test or equivalent). This approach enabled the identification and quantification of significantly up-regulated and down-regulated phosphorylated peptides between comparison groups.

### Statistics and reproducibility

At least 3 independent experimental replicates were performed for each condition, with each replicate conducted in triplicate. Statistical analyses were carried out using GraphPad Prism software (version 6). Data are presented as mean ± standard deviation (SD). The sample size (*n*) corresponds to the number of independent biological replicates. Statistical significance between groups was assessed using an unpaired Student’s *t* test. A threshold of *P* < 0.05 was considered statistically significant. Significance levels are denoted as follows: ns, not significant (*P* > 0.05); *P < 0.05; **P < 0.01; and ***P < 0.001. RNA-seq, ATAC-seq, phosphoproteomic, and metabolism experiments were performed in biological duplicate.

## Ethical Approval

C57BL/6J-*Pten^em1C^/Cya* mouse model was created by Cyagen (Suzhou, China). All mice were maintained under specific pathogen-free (SPF) conditions with access to water and food and 12-h dark/12-h light cycles. The use of mice was approved by the Animal Ethics Committee of the School of Medicine, Shandong University (Jinan, Shandong Province, China), with the approval number #2023-101.

## Data Availability

The high-throughput sequencing datasets have been deposited in the GEO database. The accession numbers are GSE301515 for scRNA-seq, GSE301159 for bulk RNA-seq, and GSE301161 for ATAC-seq. Additionally, the metabolism datasets have been submitted to the NIH Metabolomics Workbench under the accession number datatrack_id:6182. The phosphoproteomics datasets have been deposited in the PRIDE repository, with the accession number PXD065688.
