# Supplementary material for: Human Organoids Reveal PTEN-Driven Mesendoderm Specification via Retinoic Acid Signaling Suppression
Source: Research (Wash D C). 2026 Aug 14;9:1374. doi: 10.34133/research.1374 (PMC13473717; doi:10.34133/research.1374)

Human organoids reveal PTEN-driven mesendoderm specification via retinoic acid signaling suppression

Supplementary Figure 1. MES organoids generation from hiPSCs.
(**A**) IF staining of cryosectioned MES organoids derived from two additional iPSC lines (GM23720 and AICS-0060-027), confirming co-expression of EOMES and SOX17. Scale bar, 100 μm. (**B**) Quantitative PCR analysis of APS and PPS marker gene expression, demonstrating enrichment of APS markers in MES organoids derived from the iPSC lines GM23720 and AICS-0060-027. (**C**) MES organoids were harvested at multiple time points and subjected to Immunofluorescence (IF) staining with anti-SOX17 and anti-EOMES antibodies. Scale bar, 100 μm. (**D**) Temporal expression dynamics of several MES marker genes were quantitatively analyzed. (**E**) Whole-mount IF staining of MES organoids was performed to assess spatial marker distribution. Scale bar, 100 μm. (**F**) Whole-mount IF staining of endothelial cell (EC) organoids was conducted using an anti-CDH5 antibody, accompanied by phase-contrast and bright-field imaging. Scale bar, 200 μm. (**G**) 3D reconstructions of EC organoids was achieved via fluorescence microscope, Scale bar, 200 μm. Single cells dissociated from EC organoids are shown. Scale bar, 100 μm. (**H**, **I**) Temporal changes in PTEN protein levels revealed peak expression at day 2.

Supplementary Figure 2. scRNA-seq analysis of MES organoids.
(**A**, **B**) Quality assessment of scRNA-seq data included evaluation of Seurat-derived quality control metrics and visualization of doublet scores on the UMAP embedding. (**C**, **D**) Identification of APS, Epi, and CPC cell clusters, illustrated with corresponding violin plots depicting gene expression distributions. (**E**, **F**) The Sankey diagram depicts the distribution of transferred MES organoid cells across cell clusters in human and monkey embryos.

Supplementary Figure 3. *PTEN* ablation impairs MES formation and DE lineage specification. (**A**) *PTEN* was deleted in two iPSC lines (AG28869 and AICS-0060-027) using CRISPR-Cas9. *PTEN* KO-1 was derived from iPSC-AG28869, while *PTEN* KO-2 and KO-3 were generated from iPSC-AICS-0060-027. (**B**) Analysis of MES organoids derived from WT and $PTEN^{-/-}$ iPSCs (AG28869). Scale bar, 100 μm. (**C**) qPCR analysis reveals significant downregulation of MES markers including *EOMES*, *FOXA2*, *SOX17*, *GSC*, and *LHX1* in $PTEN^{-/-}$ MES organoids derived from iPSC (AG28869). (**D**) iPSC (AICS-0060-027)-derived MES organoids exhibit a marked reduction in SOX17- and EOMES-positive MES cells in the $PTEN^{-/-}$ group. Scale bar, 100 μm. (**E**) Proportion of MES cells co-expressing SOX17 and EOMES in WT and $PTEN^{-/-}$ MES organoids derived from iPSC (AICS-0060-027). (**F**) qPCR analysis reveals significant downregulation of *CYP26A1* and MES markers, including *EOMES*, *FOXA2*, *SOX17*, and *GSC* in $PTEN^{-/-}$ MES organoids derived from iPSC (AICS-0060-027). (**G**) IF staining of iPSCs-derived DE cells co-expressing *SOX17 and FOXA2*. Scale bar, 50 μm. (**H**) Quantification revealed a significant reduction in *SOX17- and FOXA2*-positive cells following *PTEN* ablation. (**I**) Expression levels of DE markers (*FOXA2*, *SOX17*) were decreased in $PTEN^{-/-}$ DE cells. (**J**) Flow cytometry analysis demonstrated a marked decrease in the proportion of DE cells upon *PTEN* deletion. (**K**) GO enrichment analysis identified key signaling pathways implicated in DE differentiation. Statistical test, two-sided *t*-test; $*P < 0.05$; $**P < 0.01$; $***P < 0.001$.

Supplementary Figure 4. *PTEN* ablation suppresses cardiac mesoderm formation.
(**A**) IF staining showed a significant reduction in cardiac progenitor cells following *PTEN* ablation. Scale bar, 100 μm. (**B**) Flow cytometry analysis demonstrated a marked decreased

in the proportions of GATA4-positive and NKX2.5-positive cells in *PTEN*$^{-/-}$ cells. (**C**) Generation of hCO from hiPSCs was performed. (**D**) IF staining confirmed expression of the CPC marker NKX2.5 in both WT and *PTEN*$^{-/-}$ hCOs. Scale bar, 100 µm. (**E**) Expression levels of cardiac mesoderm markers were significantly diminished in *PTEN*$^{-/-}$ hCOs. Statistical test, two-sided *t*-test; **P* < 0.05; ***P* < 0.01; ****P* < 0.001.

Supplementary Figure 5. EC lineage specification is impaired by *PTEN* ablation.
(**A**) A significant reduction in EC ring formation observed in the *PTEN*$^{-/-}$ group. Scale bar, 200 µm. (**B**) Phosphoproteomic profiling reveals disruption of the VEGFA–VEGFR2 signaling pathway in *PTEN*$^{-/-}$ cells. KinMap database (http://www.kinhub.org/kinmap/) was used with input of protein accession number from differentially phosphorylated proteins. The kinase families listed includes TK (tyrosine kinases), TKL (tyrosine kinase-like), CK1 (casein kinase 1), CAMK (calcium/calmodulin-dependent protein kinase), AGC (containing PKA, CMGC (containing CDKs, MAPK, GSK, CLK families), and STE (serine/threonine kinases many involved in MAPK kinases cascade).

Supplementary Figure 6. *PTEN*-OE rescues MES cell generation and convergent migration.
(**A**, **B**) *PTEN*-OE reinstated MES formation, as evidenced by the co-expression of SOX17 and EOMES. Scale bar, 100 µm. (**C**) The mRNA levels of MES marker genes, including *EOMES*, *GSC*, *FOXA2*, *LHX1*, and *SOX17*, were significantly restored in the *PTEN*-OE group. (**D**) Convergent migration of MES was markedly rescued following *PTEN*-OE. Scale bar, 50 µm. (**E**) Multidimensional scaling (MDS) analysis of transcriptomic data demonstrated separation of WT and *PTEN*$^{-/-}$ samples at both differentiation stages prior to batch correction using ComBat-seq. (**F**) ATAC-seq revealed that *PTEN* loss reduced chromatin accessibility at MES marker loci (*SOX17*, *FOXA2*) at day 3 and CPC marker loci (*MYL7*, *MYL4*) at day 6. Statistical test, two-sided *t*-test with Benjamini–Hochberg correction; **P* < 0.05; ****P* < 0.001.

Supplementary Figure 7. PTEN modulates MES cell generation via its phosphatase activity. (**A**) Western blot analysis demonstrated that the PI3K inhibitor PX-866 effectively restored MES cell generation. (**B**, **C**) The induction of MES cells was similarly rescued by PX-866 treatment, as evidence by increased MES marker expression. Scale bar, 100 µm. (**D**) Quantitative analysis showed that mRNA levels of MES-specific marker were significantly restored following PX-866 treatment. Statistical test, two-sided *t*-test with Benjamini–Hochberg correction; **P* < 0.05; ****P* < 0.001.

Supplementary Figure 8. PTEN regulates MES cell generation via CYP26A1-mediated RA signalling. (**A**) Knockdown efficiency of CYP26A1 was validated by Western blot analysis, demonstrating reduced protein levels following shRNA treatment. (**B**, **C**) *CYP26A1* knockdown significantly impaired MES cell generation. Scale bar, 100 µm. (**D**) Sanger sequencing for two *CYP26A1* deleted iPSC colonies generated by CRISPR/Cas9 system. (**E**) *CYP26A1* ablation significantly inhibited MES cell generation. Scale bar, 100 µm. (**F**) Expression levels of MES marker genes were significantly decreased in *PTEN*$^{-/-}$ MES cells. Statistical test, two-sided *t*-test; **P* < 0.05; ***P* < 0.01; ****P* < 0.001.

Supplementary Figure 9. WNT/β-catenin signalling is implicated in the PTEN-mediated regulation of MES generation. (**A**) Western blot analysis demonstrated that treatment with PI3K inhibitor PX-866 significantly restored the protein level of CYP26A1 and β-catenin. (**B**) PX-866 treatment markedly increased the mRNA level of *CYP26A1* in *PTEN*$^{-/-}$ MES cells.

(**C**) *PTEN*-OE significantly rescued *CYP26A1* expression. (**D**) Kinome dendrogram derived from human phosphoproteomics data revealed enrichment of WNT/β-catenin signaling when comparing WT and *PTEN*$^{-/-}$ MES cells. (**E**) Heatmap analysis indicated PTEN-dependent regulation and enrichment of WNT signaling components. (**F**) Western blot analysis validated the increased phosphorylation of SIRT1 in *PTEN*$^{-/-}$ MES cells. (**G**) Treatment with the SIRT1 inhibitor EX-527 significantly promoted MES cell generation compared with the DMSO-treated group in *PTEN*$^{-/-}$cells. (**H**) Quantification revealed a significant increase in *SOX17- and FOXA2*-positive cells following EX-527 treatment. (**I**) The diagram illustrates that PTEN may regulate CYP26A1 expression through the WNT/β-catenin signaling pathway. Statistical test, two-sided *t*-test with Benjamini–Hochberg correction; $*P < 0.05$; $***P < 0.001$.

Supplementary Figure 10. Excessive RA exerts a detrimental effect on MES cell generation. (**A**) Excessive RA significantly suppressed MES generation, mirroring the phenotype observed in the *PTEN*$^{-/-}$ group. (**B**) Treatment with increasing concentrations of RA resulted in a dose-dependent inhibition of MES cell generation. Scale bar, 100 μm. (**C**) Excessive RA exposure reduced the proportion of MES cells within MES organoids. Scale bar, 100 μm. (**D**, **E**) Administration of an RA antagonist enhanced MES cell generation, as evidenced by increased numbers of EOMES- and SOX17-positive cells and elevated mRNA levels of MES markers. Scale bar, 100 μm. (**F**) The RA antagonist significantly promoted MES organoid formation relative to the DMSO-treated group. Statistical test, two-sided *t*-test; $*P < 0.05$; $**P < 0.01$; $***P < 0.001$.

Supplementary Figure 1

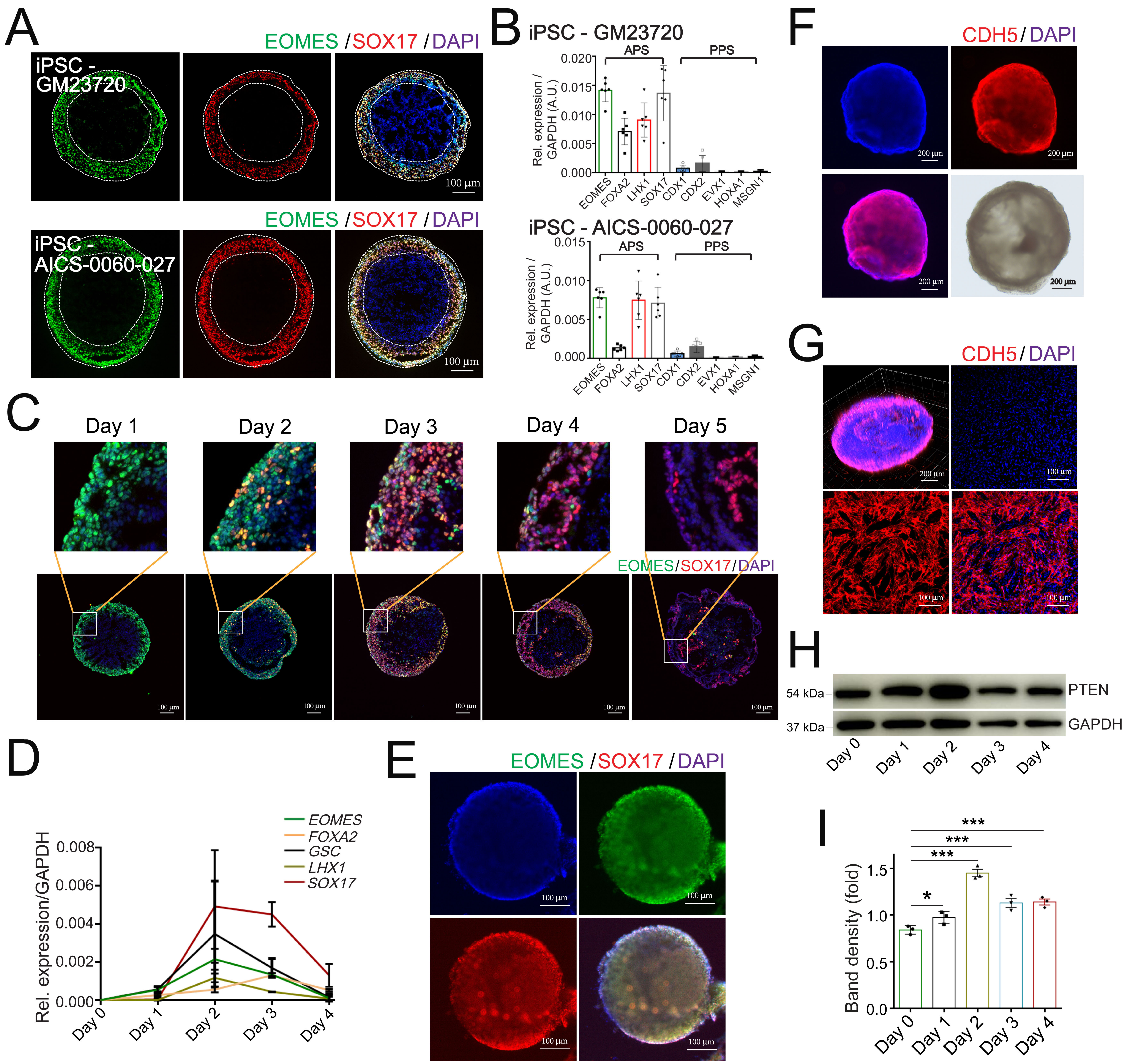

Supplementary Figure 2

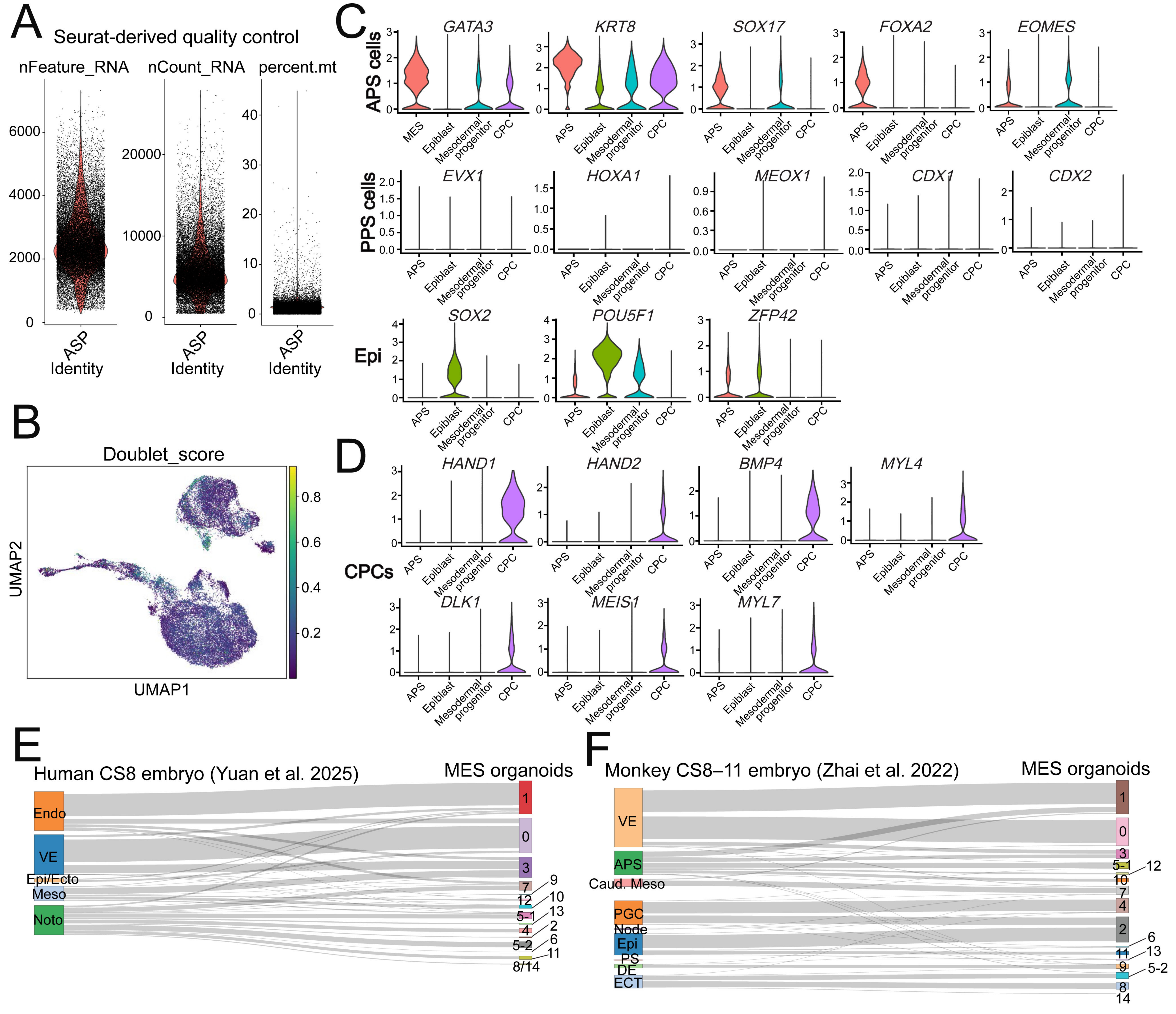

Supplementary Figure 3

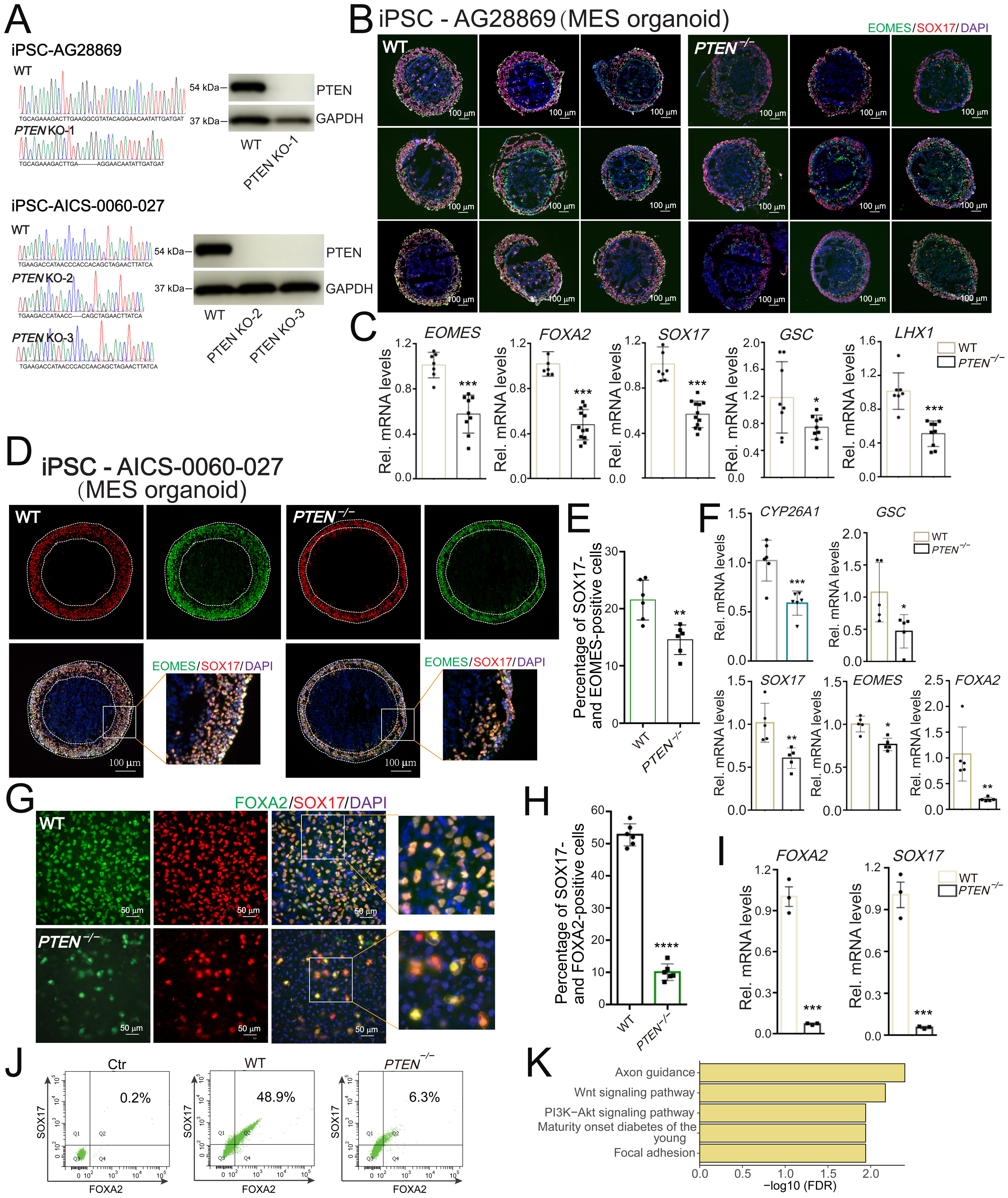

Supplementary Figure 4

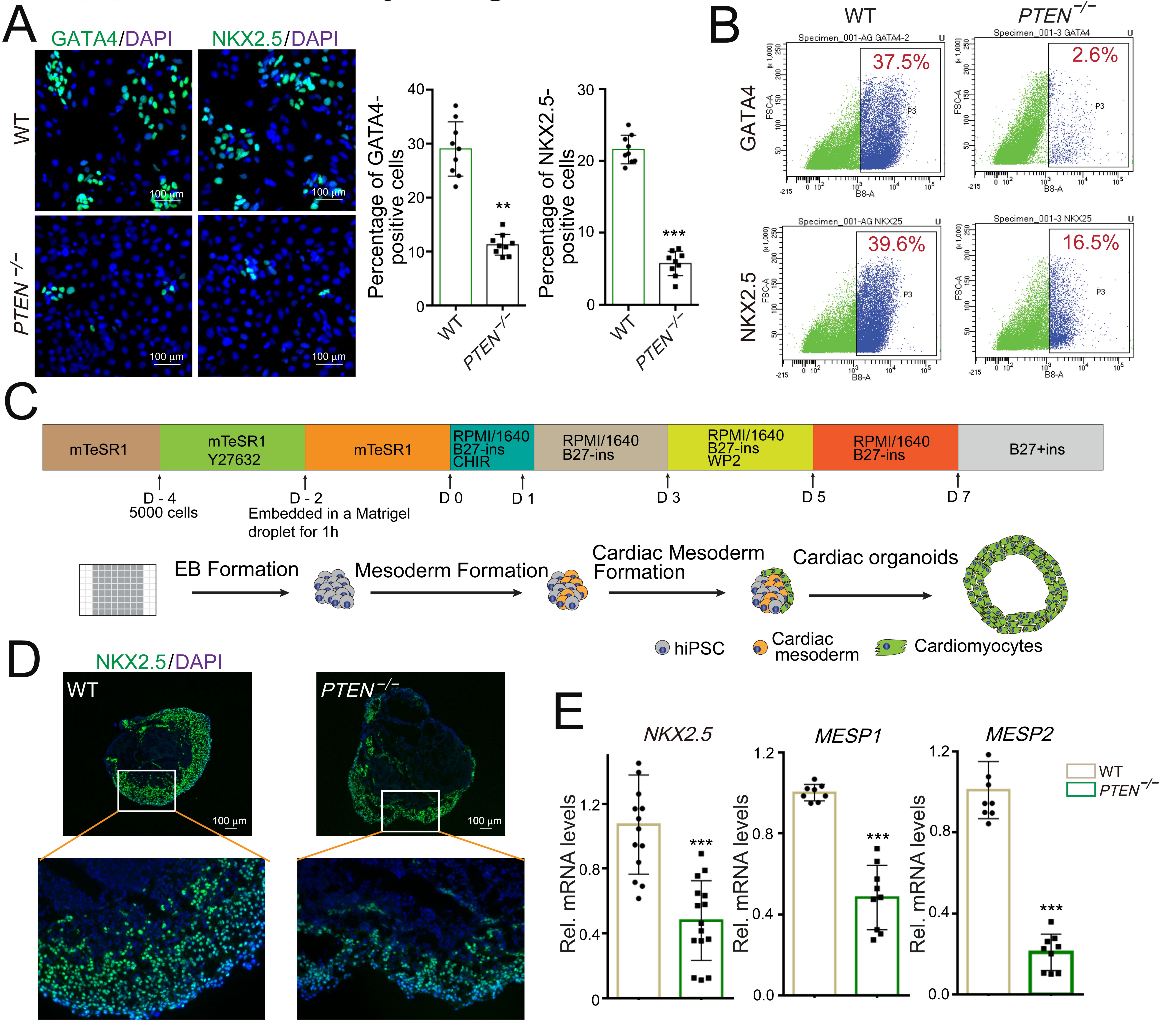

Supplementary Figure 5

A

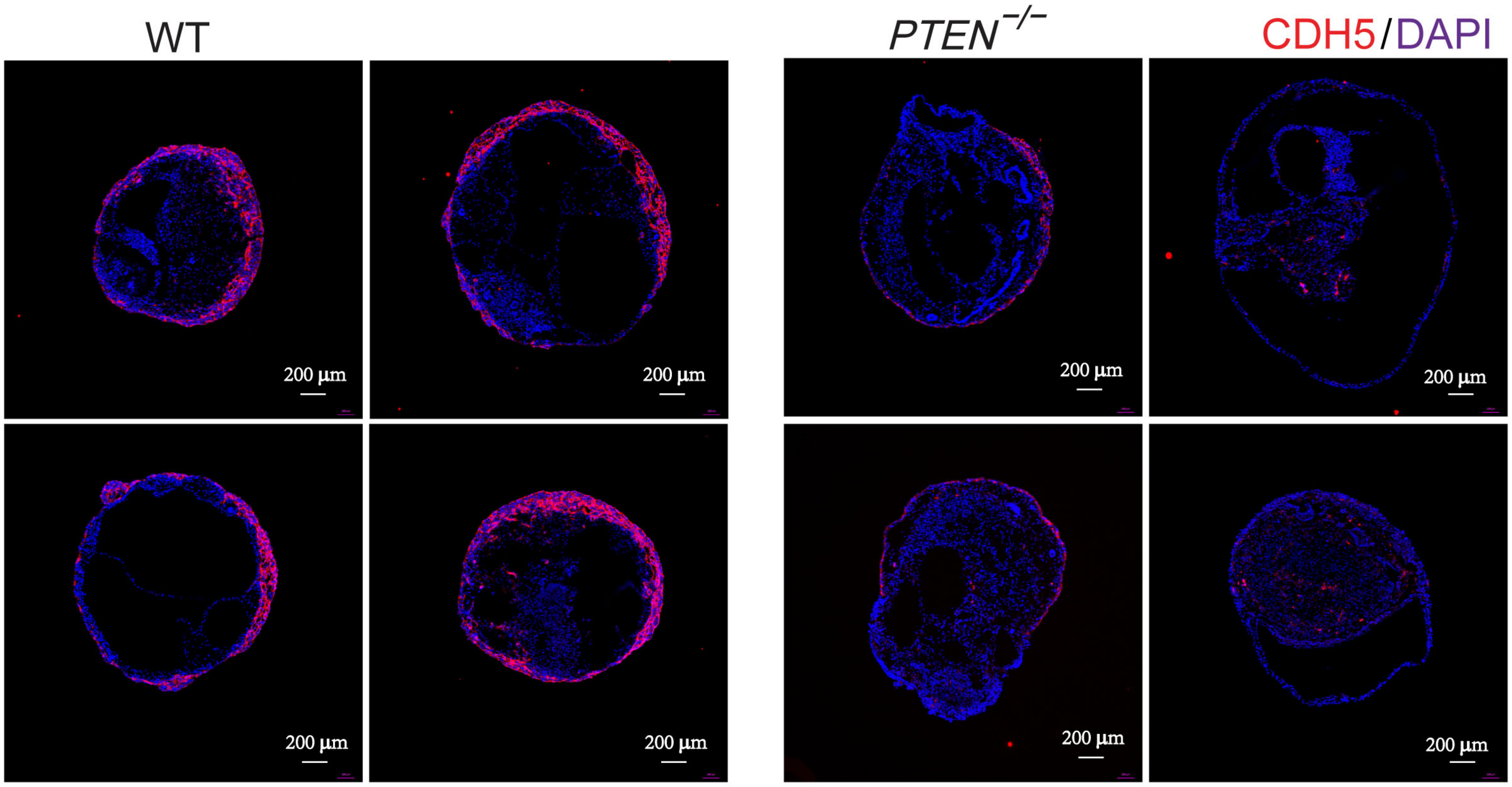

B

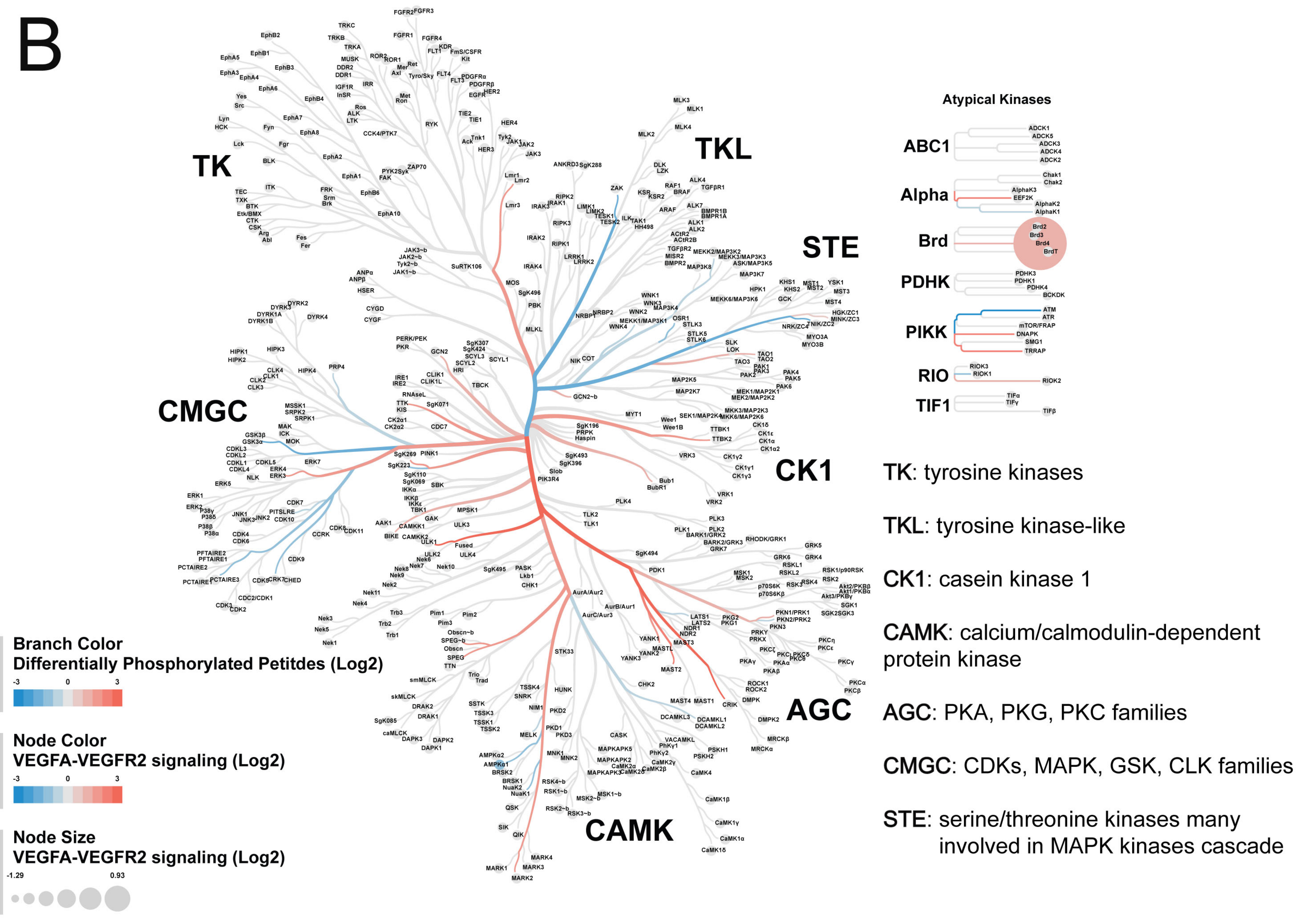

Supplementary Figure 6

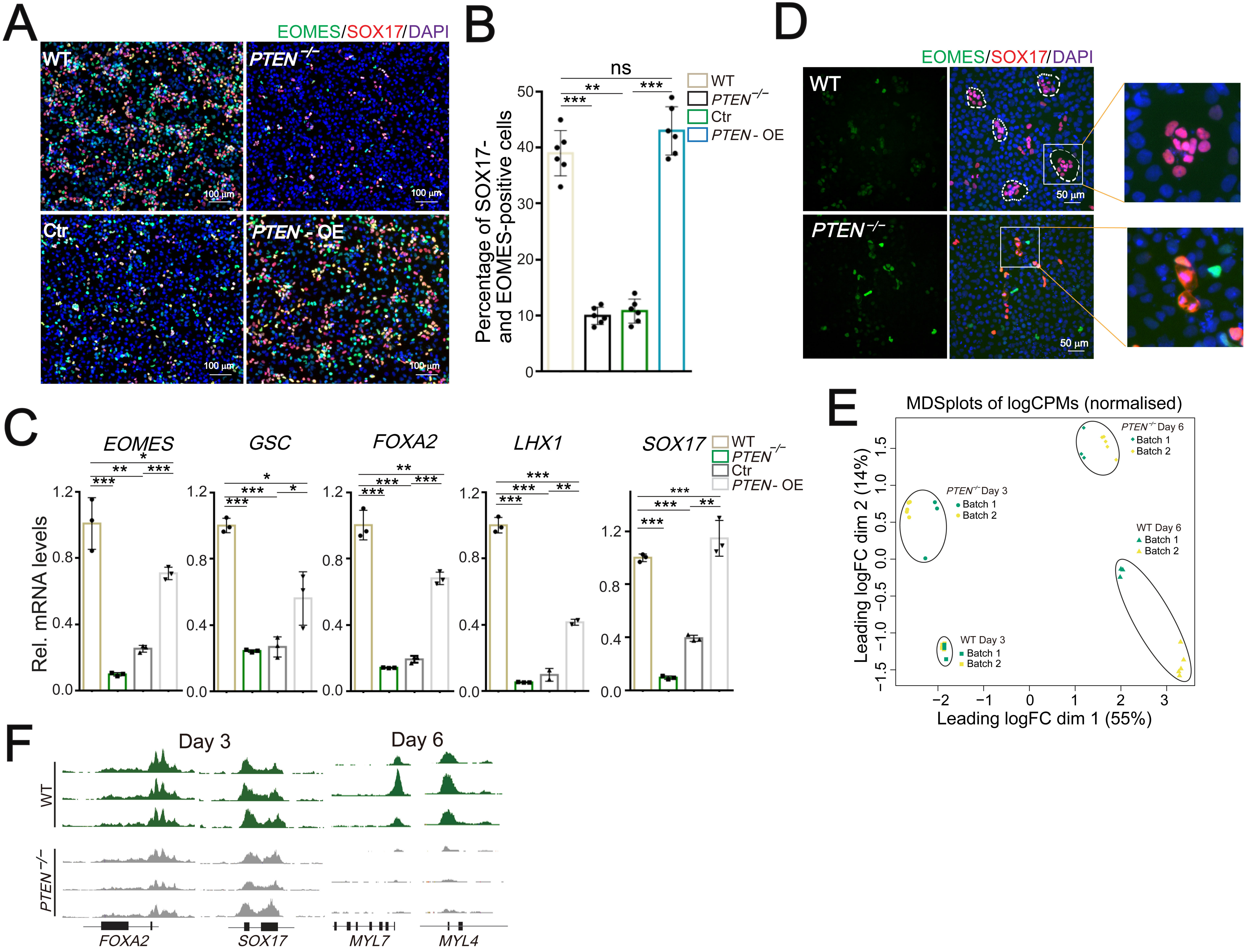

Supplementary Figure 7

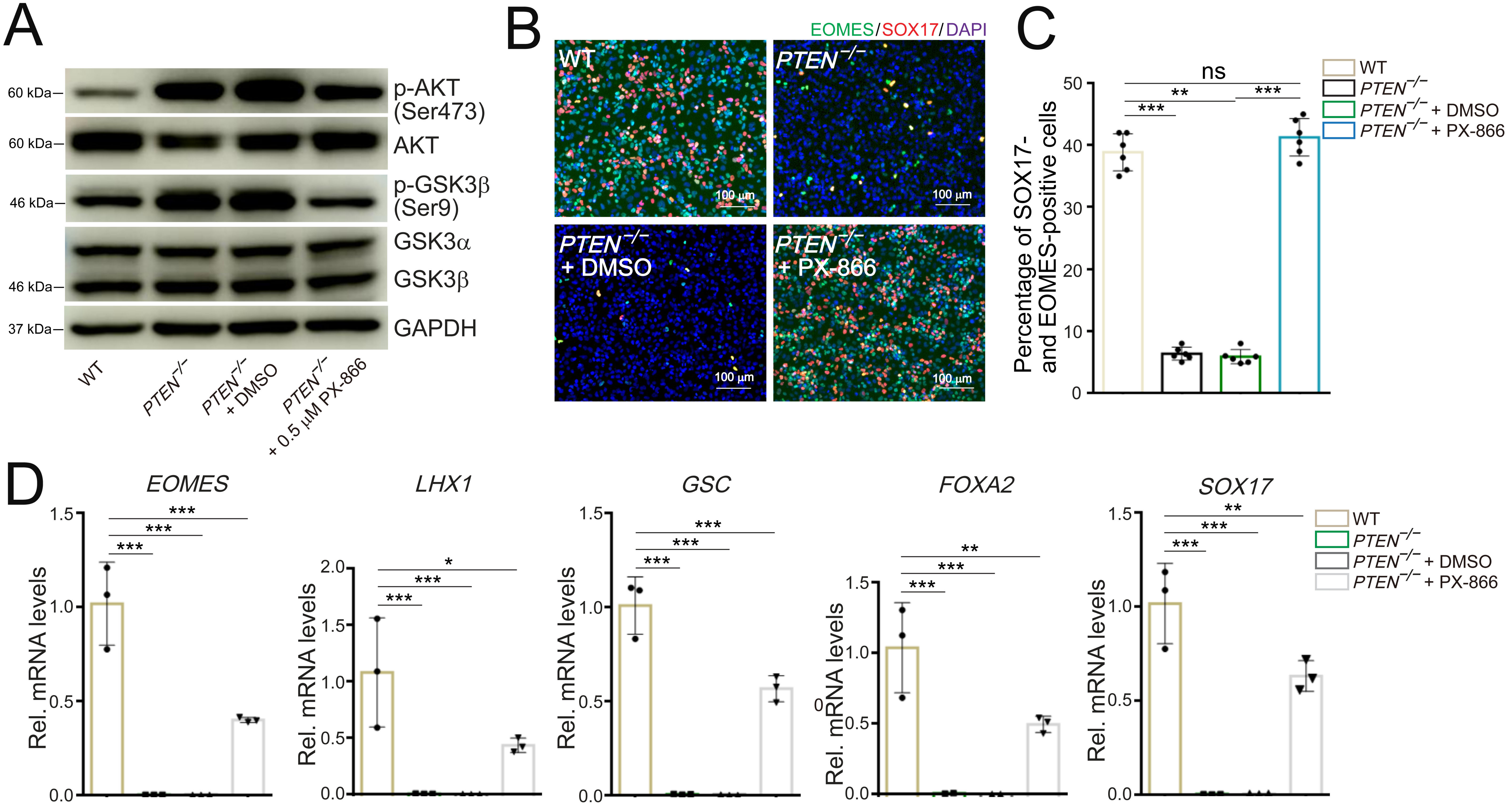

Supplementary Figure 8

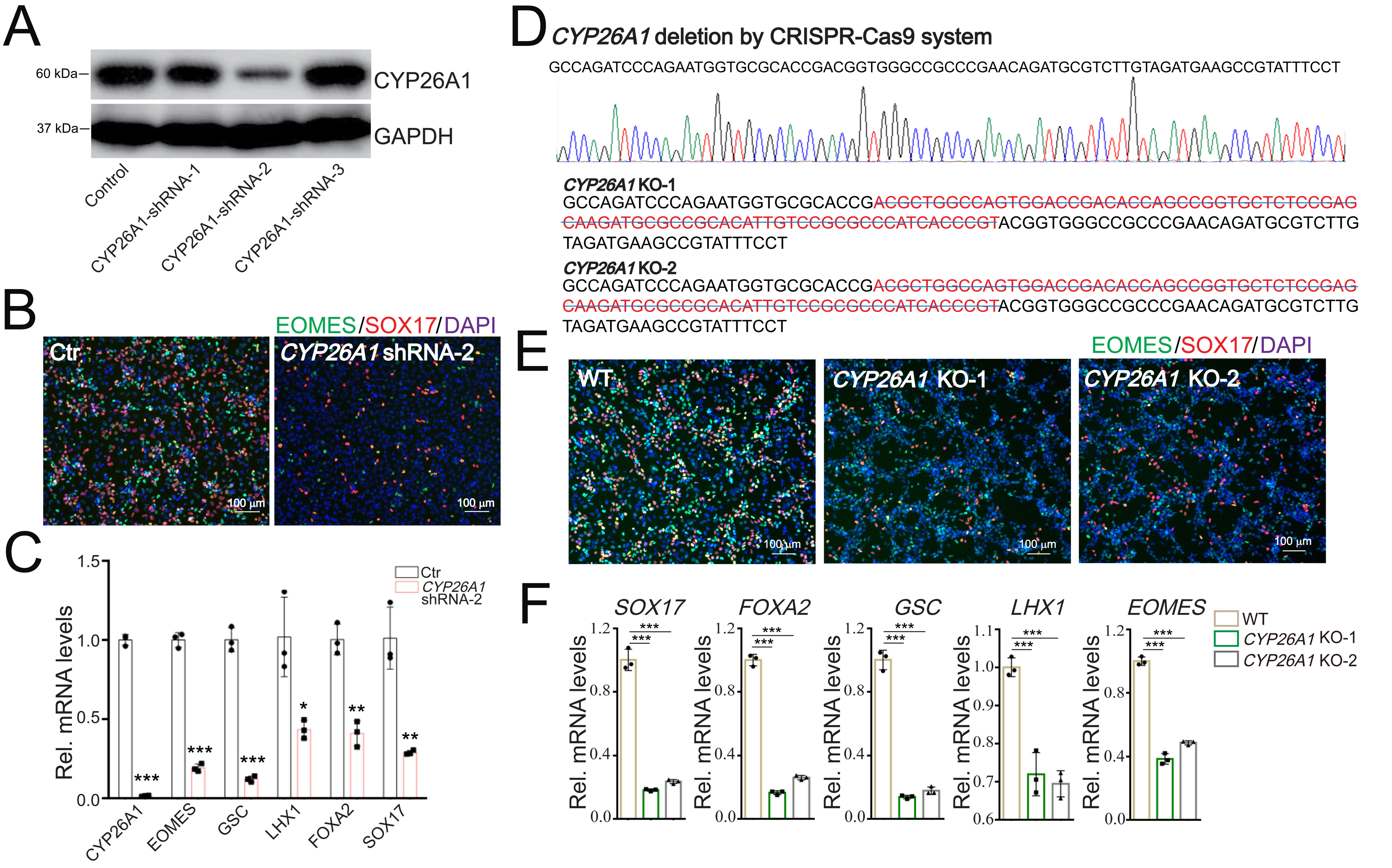

Supplementary Figure 9

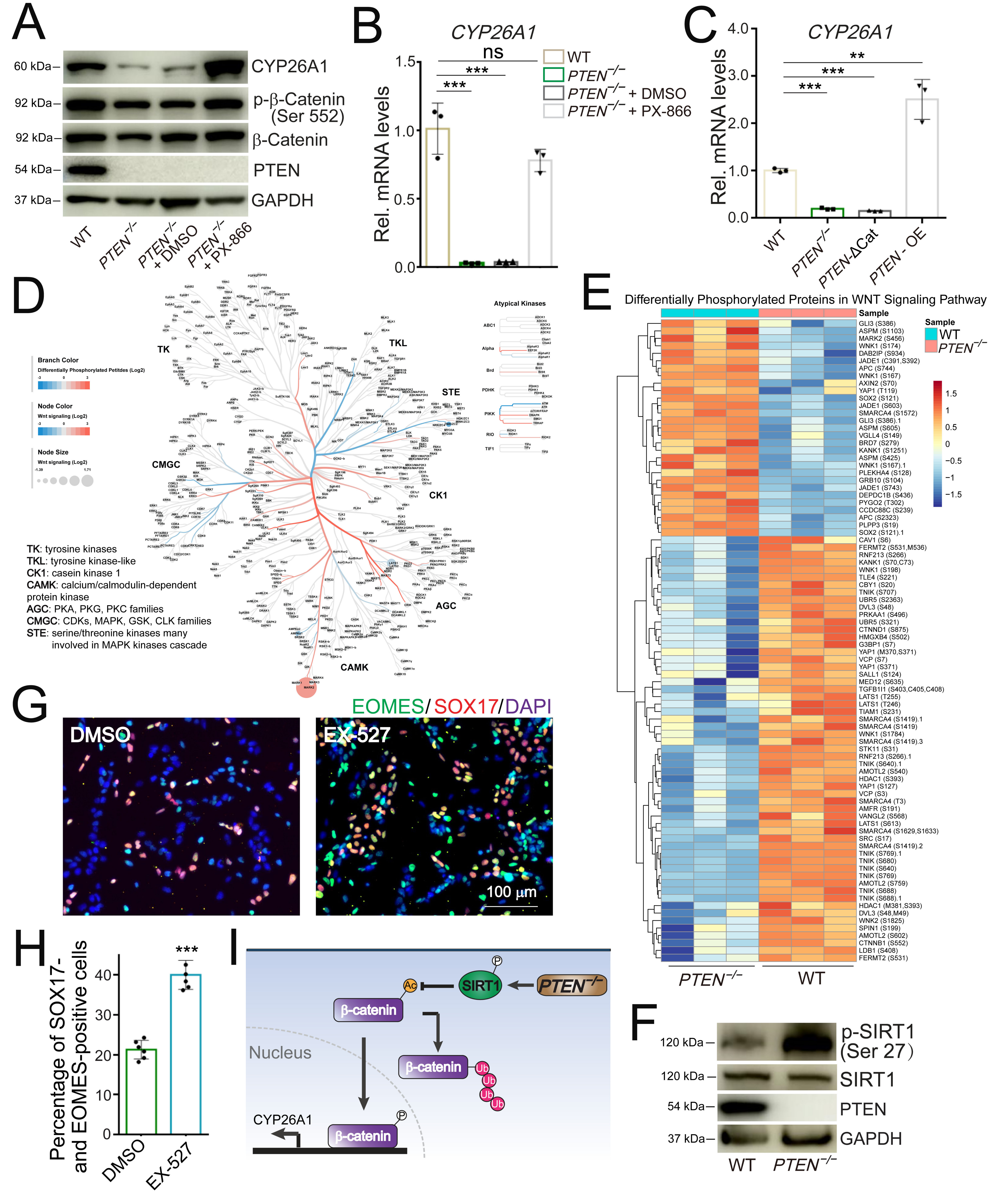

Supplementary Figure 10

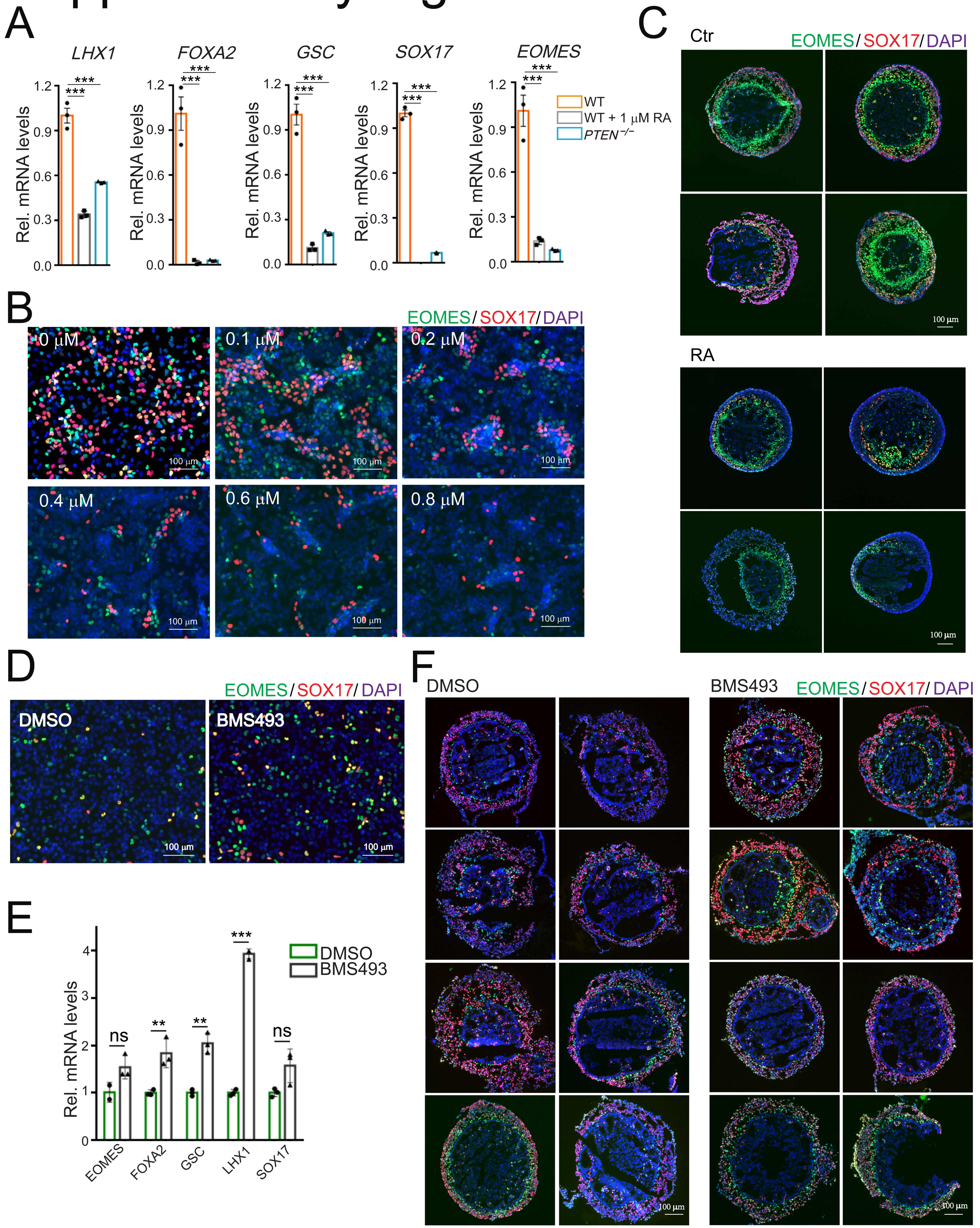

Supplement: Supplementary file 1 [file research.1374.f1.zip › Supplemental Figures-updated.pdf]
